# *gem*-Difluorination of carbon–carbon triple bonds using Brønsted acid/Bu_4_NBF_4_ or electrogenerated acid

**DOI:** 10.3762/bjoc.20.194

**Published:** 2024-09-06

**Authors:** Mizuki Yamaguchi, Hiroki Shimao, Kengo Hamasaki, Keiji Nishiwaki, Shigenori Kashimura, Kouichi Matsumoto

**Affiliations:** 1 Department of Chemistry, School of Science and Engineering, Kindai University, 3-4-1 Kowakae, Higashi-osaka, Osaka 577-8502, Japan; 2 Department of Pharmaceutical Sciences, Faculty of Pharmacy, Kindai University, 3-4-1 Kowakae, Higashi-osaka, Osaka, 577-8502, Japan

**Keywords:** carbon–carbon triple bonds, chemical method, electrochemistry, *gem*-difluorination

## Abstract

*gem*-Difluorination of carbon–carbon triple bonds was conducted using Brønsted acids, such as Tf_2_NH and TfOH, combined with Bu_4_NBF_4_ as the fluorine source. The electrochemical oxidation of a Bu_4_NBF_4_/CH_2_Cl_2_ solution containing alkyne substrates could also give the corresponding *gem*-difluorinated compounds (*in-cell* method). The *ex-cell* electrolysis method was also applicable for *gem*-difluorination of alkynes.

## Introduction

Organofluorine compounds have attracted great attention in various fields, such as organic materials and pharmaceuticals [1–3], because fluorinated compounds sometimes show specific properties [4]. So far, several methods have been developed for the synthesis of fluorinated compounds. Using nucleophilic fluorinating reagents, such as diethylaminosulfur trifluoride (DAST), HF, CsF, and AgF has been established as a reliable method. Electrophilic fluorinating reagents, such as 1-chloromethyl-4-fluoro-1,4-diazoniabicyclo[2.2.2]octane bis(tetrafluoroborate) (Selectfluor), *N*-fluorobenzenesulfonimide, and fluorobenziodoxole, are also utilized as F^+^ equivalents to introduce fluorine atoms into organic molecules. In addition, various trifluoromethylation reagents have been developed so far [5–18]. Transition-metal-catalyzed fluorination and trifluoromethylation methods have also been proposed [19–20]. Thus, the synthesis of fluorinated compounds is an active research field. Among these compounds, skeletons bearing CF_2_ units are important [21–24], because such molecules can change the physical properties and biological activity. They can also serve as building blocks for further transformations.

We have focused on the investigation of *gem*-difluorination of carbon–carbon triple bonds, because this procedure is one of the most simple but powerful and straightforward methods. In addition, there have been a few reports in the literature that seem to mainly rely on the use of HF or its complexes as a reagent. These reactions seem to proceed via the formation of the vinyl fluoride as the intermediate [25–28].

**Figure 1:**
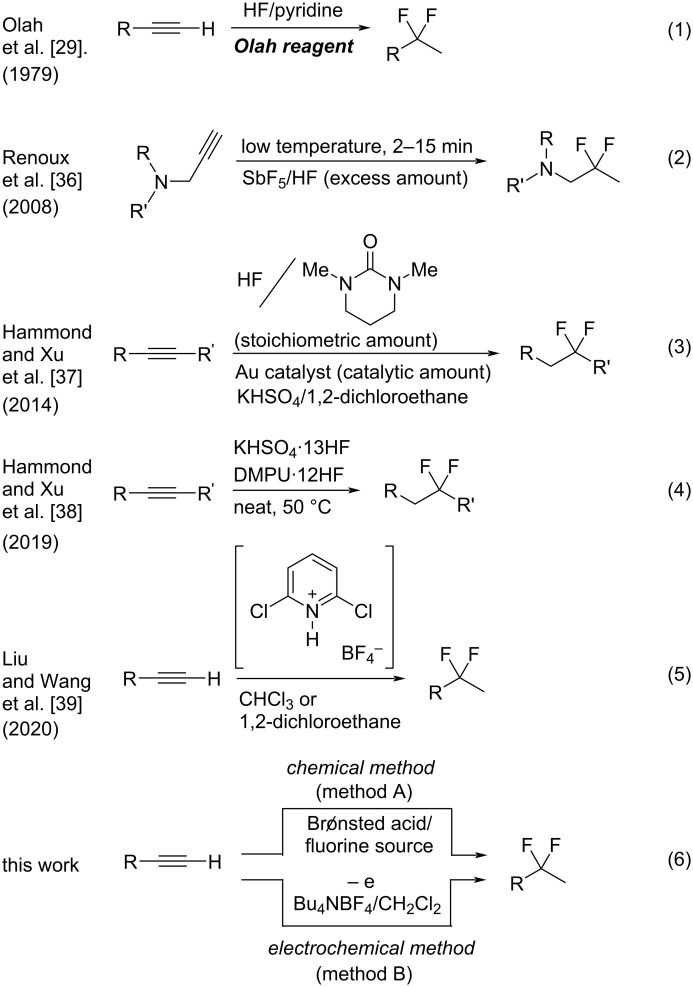
*gem*-Difluorination of carbon–carbon triple bonds. Selected examples from (1) to (5), and this work of (6).

In the first example, Olah and co-workers reported the reaction of terminal alkynes with HF/pyridine (Olah reagent) (Figure 1, reaction 1) [29–32], although the original work was developed by Linn and Plueddeman using HF [33–35]. As another example, Renoux and co-workers developed the utility of SbF_5_/HF (Figure 1, reaction 2) [36]. In 2014, the HF/*N*,*N*’-dimethylpropyleneurea (DMPU) complex in the presence of an Au catalyst was found to be a good reagent for the *gem*-difluorination of alkynes, reported by Hammond and Xu (Figure 1, reaction 3) [37]. HF/DMPU is easy to handle under experimental conditions. In addition, they recently reported the utilization of a combination of KHSO_4_·13HF and DMPU·12HF under neat conditions for the *gem*-difluorination of alkynes (Figure 1, reaction 4) [38]. In 2020, the utility of 2,6-dichloropyridinium tetrafluoroborate was nicely demonstrated for the *gem*-difluorination by Liu and Wang (Figure 1, reaction 5) [39].

Although some procedures have been reported, the use of hazardous reagents such as HF is still inevitable [40–41]. Quite recently, Crimmin and co-workers reported *gem*-difluorination by shuttling between fluoroalkanes and alkynes, in which catalytic HF played a key role [42]. In the course of our study on the fluorination reaction, we have envisioned that the combination of a Brønsted acid, such as Tf_2_NH and TfOH, with Bu_4_NBF_4_ might be effective to promote the *gem*-difluorination of alkynes because of the in situ generation of HF equivalents (Figure 1, reaction 6, chemical method). In addition, the electrogenerated acid (EGA) [43–52] from a solution of Bu_4_NBF_4_/CH_2_Cl_2_ containing substrates might also promote the same reactions (Figure 1, reaction 6, electrochemical method). Currently, electrochemistry can be regarded as a promising technique in organic synthesis, because heavy-metal reagents can be avoided for the oxidation or reduction of organic molecules. Herein, we wish to report that the combination of the excess amount of Brønsted acid and Bu_4_NBF_4_ or the use of an EGA in Bu_4_NBF_4_/CH_2_Cl_2_ can serve as suitable reagents for the *gem*-difluorination of alkynes. These procedures are practical, simple and environmentally friendly, because HF or its equivalent is indirectly prepared and utilized in only solution phase.

## Results and Discussion

First, we have chosen hex-5-yn-1-ylbenzene (**1a**) as the model substrate in the reaction optimization (Table 1, method A). The reaction was carried out as follows: Hex-5-yn-1-ylbenzene (**1a**, 0.5 mmol) was reacted with the Brønsted acid (*X* equiv) and the fluorine source (*Y* equiv) in the solvent (4 mL) at temperature of *T* (°C) for *Z* hours. The chemical yield of the desired product, (5,5-difluorohexyl)benzene (**2a**), was evaluated for reaction optimization by using the ^19^F nuclear magnetic resonance (NMR) yield, in which trifluorotoluene (CF_3_C_6_H_5_) was used as an internal standard. The use of Tf_2_NH (5 equiv or 10 equiv) and Bu_4_NBF_4_ (5 equiv) in CH_2_Cl_2_ at room temperature gave the corresponding product **2a** in up to 83% yield (Table 1, entries 1–5). The use of CF_3_COOH did not yield **2a** at all (Table 1, entry 6), but TfOH gave the product **2a** in 72% yield (Table 1, entry 7). As for the solvent, CH_3_CN slightly afforded **2a**, although *N*,*N*-dimethylformamide (DMF) and dimethyl sulfoxide (DMSO) were not suitable for the reactions (Table 1, entries 8–10). A fluorine source, such as Bu_4_NF or BF_3_·Et_2_O, instead of Bu_4_NBF_4_ was not effective (Table 1, entries 11 and 12). Finally, investigations of the amount of Bu_4_NBF_4_ and the reaction temperature demonstrated that conditions including Bu_4_NBF_4_ (9 equiv) and room temperature gave the best result (Table 1, entries 13–17). Based on the above investigation, we decided to use the optimized conditions in entry 2, because reducing the amount of Bu_4_NBF_4_, for example, to 5 equiv is important from the viewpoint of eco-friendly chemical synthesis. The reaction time of 16 h also seems to be suitable for the investigation. Thus, the combination of Tf_2_NH and Bu_4_NBF_4_ might generate HBF_4_ in the solution.

**Table 1:** Optimization of the *gem*-difluorination of hex-5-yn-1-ylbenzene (**1a**) to form difluorinated compound **2a** (method A).

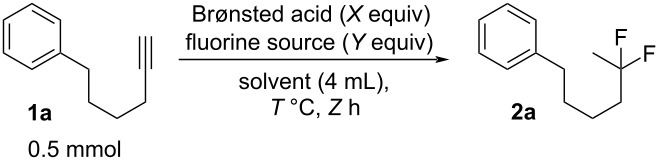								

Entry	Brønsted acid	*X* (equiv)	Fluorine source	*Y* (equiv)	Solvent	Reaction time *Z* (h)	*T* (°C)	% Yield^a^

1	Tf_2_NH	5	Bu_4_NBF_4_	5	CH_2_Cl_2_	8	rt	83
2	Tf_2_NH	5	Bu_4_NBF_4_	5	CH_2_Cl_2_	16	rt	83 (72)^b^
3	Tf_2_NH	5	Bu_4_NBF_4_	5	CH_2_Cl_2_	24	rt	82
4	Tf_2_NH	3	Bu_4_NBF_4_	5	CH_2_Cl_2_	16	rt	75
5	Tf_2_NH	10	Bu_4_NBF_4_	5	CH_2_Cl_2_	16	rt	46
6	CF_3_COOH	5	Bu_4_NBF_4_	5	CH_2_Cl_2_	16	rt	n.r.^c^
7	TfOH	5	Bu_4_NBF_4_	5	CH_2_Cl_2_	16	rt	72
8	Tf_2_NH	5	Bu_4_NBF_4_	5	CH_3_CN	16	rt	6
9	Tf_2_NH	5	Bu_4_NBF_4_	5	DMF	16	rt	n.r.^c^
10	Tf_2_NH	5	Bu_4_NBF_4_	5	DMSO	16	rt	n.r.^c^
11^d^	Tf_2_NH	5	Bu_4_NF	5	CH_2_Cl_2_	16	rt	n.r.^c^
12	Tf_2_NH	5	BF_3_·Et_2_O	5	CH_2_Cl_2_	16	rt	n.d.^e^
13	Tf_2_NH	5	Bu_4_NBF_4_	3	CH_2_Cl_2_	16	rt	50
14	Tf_2_NH	5	Bu_4_NBF_4_	9	CH_2_Cl_2_	16	rt	89
15	Tf_2_NH	5	Bu_4_NBF_4_	9	CH_2_Cl_2_	16	0	31
16	Tf_2_NH	5	Bu_4_NBF_4_	9	CH_2_Cl_2_	16	−40	n.r.^c^
17	Tf_2_NH	5	Bu_4_NBF_4_	9	CH_2_Cl_2_	16	40	82

^a19^F NMR yields. Trifluorotoluene (CF_3_C_6_H_5_) was used as an internal standard. ^b^Isolated yield after silica gel column chromatography of crude product. ^c^n.r. = no reaction. ^d^A solution of Bu_4_NF/THF underwent vacuum drying to prepare Bu_4_NF without THF. Then, CH_2_Cl_2_ was added to Bu_4_NF to prepare a solution. ^e^n.d. = not detected.

**Scheme 1:**
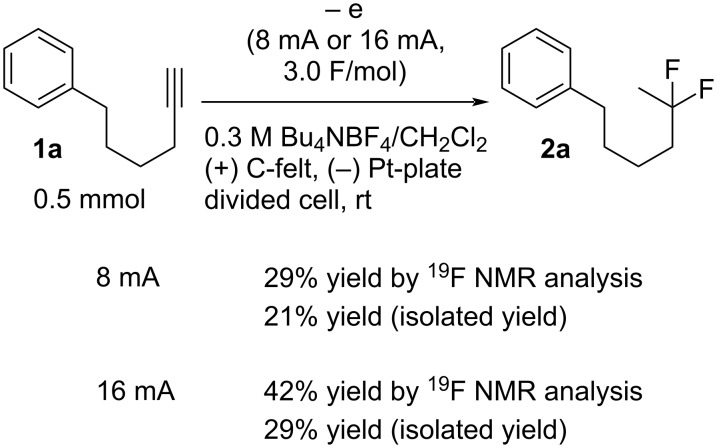
*gem*-Difluorination promoted by electrogenerated acids (method B).

Next, the electrochemical method was studied for *gem*-difluorination. In a previous report by us, we found that the electrogenerated acids of “H^+^BF_4_^−^” equivalents can actually serve as H^+^ equivalents [51–52]. We have envisioned that electrogenerated acids such as ’’H^+^BF_4_^−^’’ equivalents might serve as good reagents for the *gem*-difluorination of alkynes. Thus, we have examined the electrochemical oxidation of a solution of Bu_4_NBF_4_/CH_2_Cl_2_ containing **1a** (0.5 mmol) in a divided cell using 8 mA or 16 mA (Scheme 1, method B, *in-cell* method). *In-cell* method means that EGA was generated in the presence of the substrate. The total electricity of 3.0 F/mol vs **1a** was passed to the solution. Interestingly, the result gave the corresponding difluorinated compound **2a** in 29% yield in the case of 8 mA, as shown by ^19^F NMR analysis. In addition, **2a** was obtained in 42% yield by ^19^F NMR analysis in the case of 16 mA [53].

With the successful formation of (5,5-difluorohexyl)benzene (**2a**) by the chemical (method A) and electrochemical oxidation (method B) methods in hand, we have investigated the scope and limitations of *gem*-difluorination for various alkynes (Table 2). Electrochemical oxidation of method B was conducted by using 8 mA. The reaction of but-3-yn-1-ylbenzene (**1b**) in method A gave the corresponding compound **2b** in 21% isolated yield (Table 2, entry 1). The ^19^F NMR result indicated 63% yield. Because of the low molecular weight of **2b**, the isolated yield might be somewhat lower. In contrast, method B produced **2b** in 6% isolated yield (Table 2, entry 2). The ^19^F NMR result indicated 29% yield. As for the internal carbon–carbon triple bonds, diphenylacetylene (**1c**) was tested, but the desired product **2c** was not obtained in any of the two methods (Table 2, entries 3 and 4). In the case of an aliphatic terminal alkyne, such as dec-1-yne (**1d**), the ^19^F NMR study indicated 46% yield with method A (Table 2, entry 5), but it was difficult to purify and isolate product **2d** because of the low molecular weight. Scale up conditions of method A, for the purpose of the isolation, led to the formation of the corresponding product **2d** in 40% yield as the ^19^F NMR analysis (Table 2, entry 6), but the isolation of **2d** was difficult [54]. Method B gave **2d** in 35% yield, as shown by the ^19^F NMR analysis (Table 2, entry 7). Another alkyne, namely, octadec-1-yne (**1e**), was found to be a nice substrate for *gem*-difluorination to yield the difluorinated compound **2e** (Table 2, entries 8 and 9). Interestingly, terminal alkynes bearing –OH and –O– functional groups, such as **1f** and **1g**, were used for reactions, and the corresponding products **2f** and **2g** were obtained by both methods (Table 2, entries 10–13). In addition, 2-(pent-4-yn-1-yl)isoindoline-1,3-dione (**1h**) was utilized for the construction of the CF_2_ unit under the same conditions to give **2h** (Table 2, entries 14 and 15). The substrate bearing a halogen, such as 10-iododec-1-yne (**1i**) in method A, produced the corresponding **2i** in 60% isolated yield (Table 2, entry 16). Likewise, method B also gave **2i** in 21% yield, as shown by the ^19^F NMR analysis (Table 2, entry 17), but it was difficult to purify and isolate the product **2i** in this case. Finally, the internal aliphatic alkyne such as dodec-6-yne (**1j**) was found to be effective for the *gem*-difluorination. Method A gave **2j** in 38% isolated yield, and method B produced **2j** in 10% isolated yield (Table 2, entries 18 and 19).

**Table 2:** Scope and limitations.

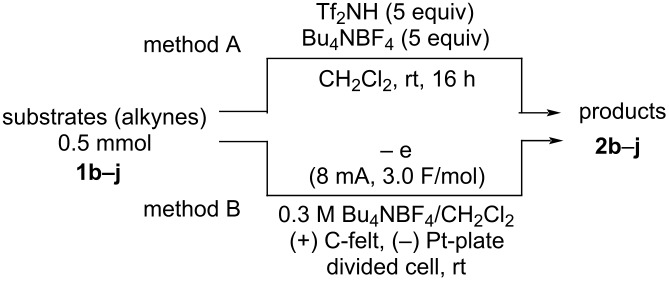				

Entry	Substrate	Product	Method	% Yield^a^

1	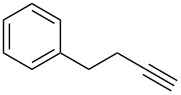 **1b**	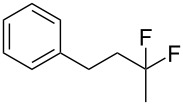 **2b**	A	21 (63)
2			B	6 (29)
3	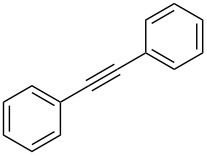 **1c**	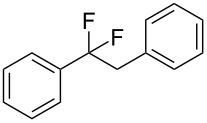 **2c**	A	n.d.^b^
4			B	n.d.^b^
5	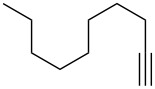 **1d**	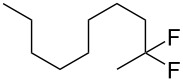 **2d**	A	–^c^ (46)
6			A	4^c^ (40)^d^
7			B	–^c^ (35)
8	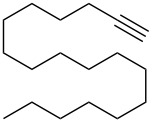 **1e**	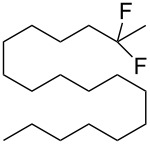 **2e**	A	67 (86)
9			B	50^e^ (45)
10	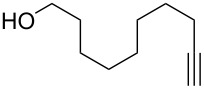 **1f**	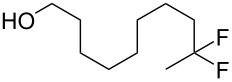 **2f**	A	47 (59)
11			B	41^e^ (37)
12	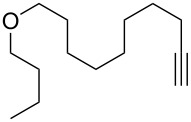 **1g**	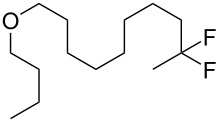 **2g**	A	58^e^ (70)
13			B	40^e^ (46)
14^f^	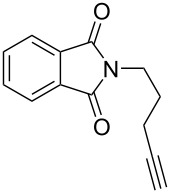 **1h**	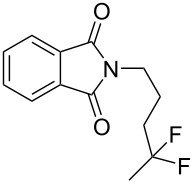 **2h**	A	37 (55)
15			B	15 (13)
16	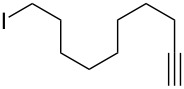 **1i**	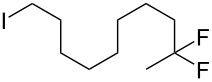 **2i**	A	60 (81)
17			B	–^c^ (21)
18	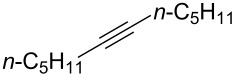 **1j**	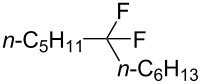 **2j**	A	38 (62)
19			B	10 (24)

^a^Isolated yields. Silica gel column chromatography and/or preparative GPC separation were/was conducted for the purification. ^19^F NMR yields of the crude products are shown in parentheses. ^b^n.d. = not detected. ^c^It was impossible to purify and isolate the corresponding product, although the product was confirmed by NMR analysis, when the crude product was prepared. The reason might be due to volatility derived from the low molecular weight. ^d^The reaction was conducted in the fourfold scale. ^e^Isolated products contained a small amount of impurity. ^f^The conditions such as Bu_4_NBF_4_ (9 equiv) and Tf_2_NH (5 equiv) in CH_2_Cl_2_ at 40 °C for 16 h were used.

**Scheme 2:**
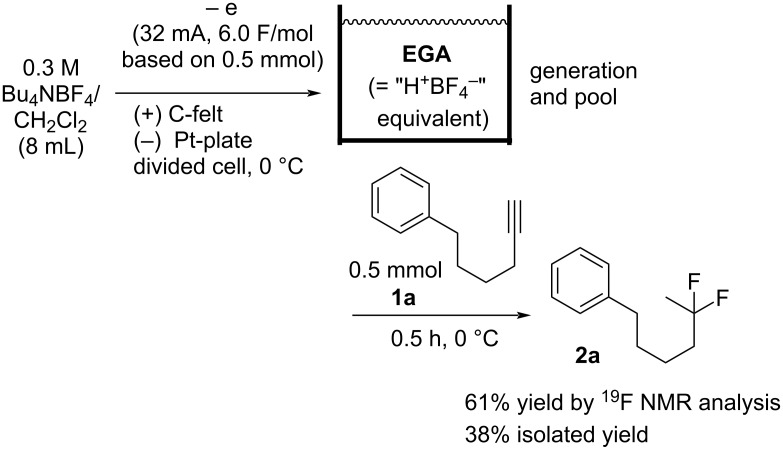
Generation and accumulation of EGA followed by the reaction with **1a** for **2a**.

Another procedure involving electrochemical oxidation was also applied (the *ex-cell* method) [55–56]. *Ex-cell* method means that EGA was generated in the absence of the substrate, and the substrate was added to the solution after the electrolysis. Optimized conditions and the result are described in Scheme 2. Namely, the electrochemical oxidation of a 0.3 M Bu_4_NBF_4_/CH_2_Cl_2_ solution (8 mL) at 0 °C using 32 mA generated and accumulated the EGA as the pool. An electricity of 6.0 F/mol based on 0.5 mmol was passed to the solution. In order to suppress the increase of the solution temperature under the electrolysis, the electrolysis was conducted at 0 ^o^C. Then, the solution containing EGA was allowed to react with **1a** (0.5 mmol) at 0 °C for 0.5 h, giving the corresponding product **2a** in 61% yield, as demonstrated by the ^19^F NMR analysis. The result indicated that CH_2_Cl_2_ of the solvent might be oxidized and H^+^ species (or equivalent units) might be generated by the electrolysis in this case. In addition, *ex-cell* method can avoid the over-oxidation of **2a**, although the excess electricity was passed to the solution.

**Scheme 3:**
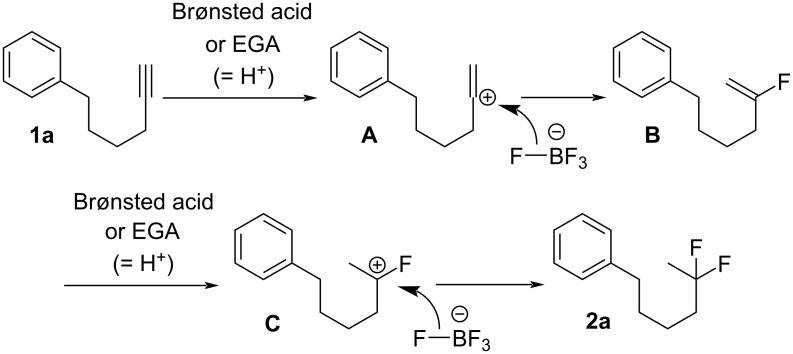
Plausible reaction mechanism.

A plausible reaction mechanism for the current reactions is described in Scheme 3. The reaction of carbon–carbon triple bonds and H^+^ species, which are derived from the Brønsted acid (in method A) or EGA (in method B), gives the vinylic carbocation intermediate **A**, which can react with BF_4_^−^ to give fluorinated alkene **B** [57–60]. In the next step, **B** can undergo the second addition of H^+^, followed by the incorporation of F^−^ into the carbocation intermediate **C**, forming the difluorinated compound **2a**. The carbocation adjacent to the F atom might be stabilized by the unshared electron pair of F. Thus, the chemical and electrochemical methods we developed here seem to be superior to the conventional method, because the chemical method requires a usual Brønsted acid and solid Bu_4_NBF_4_, which can avoid the use of dangerous HF solutions. The electrochemical method also needs only electricity and solid Bu_4_NBF_4_, which realizes in situ formation of “HBF_4_” equivalents.

## Conclusion

In summary, we have carried out the *gem*-difluorination of carbon–carbon triple bonds using Tf_2_NH/Bu_4_NBF_4_ or EGA from Bu_4_NBF_4_/CH_2_Cl_2_. The feature superiority of these methods is that they do not directly require the use of hazardous HF reagents and expensive metal catalysts. The simple combination of a Brønsted acid with Bu_4_NBF_4_ as the fluorine source as well as a simple electrolysis in Bu_4_NBF_4_/CH_2_Cl_2_ represent new routes to synthesize CF_2_-incorporated organic molecules from alkynes. Further synthetic applications are in progress in our laboratory.

## Supporting Information

File 1 Experimental procedure, characterization data of compounds and copies of spectra of ^1^H NMR and ^13^C NMR.

## Data Availability

All data that supports the findings of this study is available in the published article and/or the supporting information to this article.
