# Supplementary material for: gem-Difluorination of carbon–carbon triple bonds using Brønsted acid/Bu4NBF4 or electrogenerated acid
Source: Beilstein J Org Chem. 2024 Sep 6;20:2261–9. doi: 10.3762/bjoc.20.194 (PMC11403803; doi:10.3762/bjoc.20.194)
Supplement: File 1 — Experimental procedure, characterization data of compounds and copies of spectra of 1H NMR and 13C NMR. [file Beilstein_J_Org_Chem-20-2261-s001.pdf]

BEILSTEIN JOURNAL OF ORGANIC CHEMISTRY

Supporting Information

for

gem-Difluorination of carbon–carbon triple bonds using Brønsted acid/Bu_4NBF_4 or electrogenerated acid

Mizuki Yamaguchi, Hiroki Shimao, Kengo Hamasaki, Keiji Nishiwaki, Shigenori Kashimura and Kouichi Matsumoto

Beilstein J. Org. Chem. **2024,** *20,* 2261–2269. doi:10.3762/bjoc.20.194

Experimental procedure, characterization data of compounds and copies of spectra of ^{1}H NMR and ^{13}C NMR

License and Terms: This is a supporting information file under the terms of the Creative Commons Attribution License (https://creativecommons.org/licenses/by/4.0). Please note that the reuse, redistribution and reproduction in particular requires that the author(s) and source are credited and that individual graphics may be subject to special legal provisions.
The license is subject to the *Beilstein Journal of Organic Chemistry* terms and conditions: (https://www.beilstein-journals.org/bjoc/terms)

General remarks.

^{1}H and ^{13}C NMR spectra were measured by using a MERCURY 300 (^{1}H, 300 MHz; ^{13}C, 75 MHz), and a JEOL JNM-ECS 400 (^{1}H, 400 MHz; ^{13}C, 100 MHz; ^{19}F, 376 MHz) spectrometer. Unless otherwise noted, $CDCl_3$ was used as NMR solvent. The chemical shifts of ^{1}H NMR in $CDCl_3$ are reported using 7.26 ppm from the residual $CHCl_3$ or 0.00 ppm from tetramethylsilane (Me_4Si) as an internal standard. The chemical shifts of ^{13}C NMR in $CDCl_3$ are reported using 77.0 ppm. The chemical shifts of ^{19}F NMR in $CDCl_3$ are reported using −62.7 ppm of trifluorotoluene ($CF_3C_6H_5$) as an internal standard. After the preparation of the crude product, ^{19}F NMR spectrum was measured in the presence of trifluorotoluene ($CF_3C_6H_5$) as an internal standard. After the isolation and purification, the isolated material was also measured by ^{19}F NMR to confirm the purity and absence of trifluorotoluene ($CF_3C_6H_5$) of an internal standard. In this supporting information, ^{19}F NMR spectra at the crude product were shown below, unless mentioned. ^{1}H and ^{13}C NMR spectra were also showed, which were after the isolation and purification.

Mass spectra (HRMS) were measured by a Thermo Fisher Scientific EXACTIVE Plus spectrometer. GC analysis was carried out by using a Shimadzu GC 2014 apparatus, equipped with a capillary column and FID. GC–MS (EI) analysis was carried out by using an Agilent 6890N apparatus, equipped with an Agilent 5973N Mass Selective Detector and/or an Agilent 7890A Mass Selective Detector, equipped with an Agilent 5975C inert XL MSD with Triple Axis Detector. Merck pre-coated silica gel F_{254} plates (thickness 0.25 mm) were used for the TLC analysis. A silica gel column (Kanto Chem. Co., Silica Gel N, spherical, neutral, 40–100 μm) was used for the flash chromatography by using an air pump. Preparative GPC separation was carried out by using LC-9201, LC-9210 NEXT, or LC-9110 NEXT equipped with JAIGEL-1H and JAIGEL-2H, in which $CHCl_3$ was used as an eluent. All reactions were carried out under N_2 atmosphere, unless mentioned.

Materials.

CH_2Cl_2 containing a small amount of MeOH as the stabilizer was purchased from SASAKI Chemical Co. and Sigma-Aldrich Japan. Dried and pure CH_2Cl_2 was prepared according to reported procedure.[1] Bu_4NBF_4 was purchased from commercial suppliers, and dried at 50 °C under 1 mmHg overnight to remove a small amount of water. The substrates such as but-3-yn-1-ylbenzene (**1b**), diphenylacetylene (**1c**), dec-1-yne (**1d**), octadec-1-yne (**1e**), dec-9-yn-1-ol (**1f**), 2-(pent-4-yn-1-yl)isoindoline-1,3-dione (**1h**), and dodec-6-yne (**1j**) were purchased from commercial suppliers and used without further purification. Unless otherwise noted, all materials were obtained from commercial suppliers and used without further purification.

Synthesis of hex-5-yn-1-ylbenzene (1a).

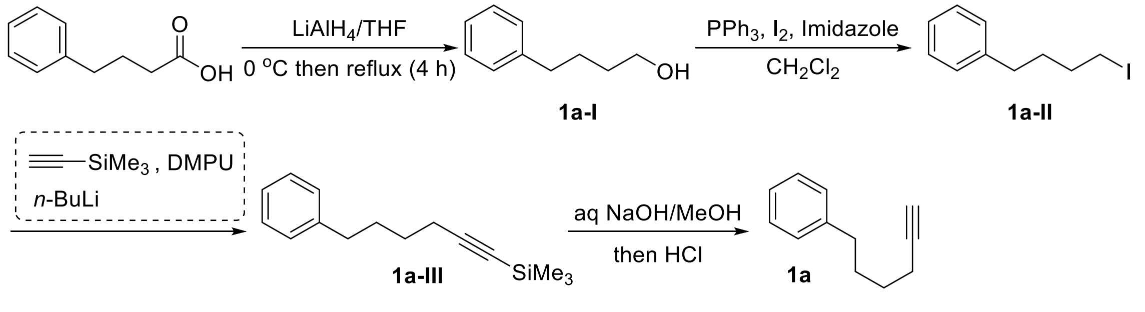

To the suspension of $LiAlH_4$ (1.54 g, 40.6 mmol) in THF (100 mL) at 0 °C was slowly added a solution of 4-phenylbutanoic acid (6.58 g, 40.1 mmol)/THF (32 mL), and reaction temperature was increased to room temperature. Then, the solution was refluxed for 4 h. After the reaction, temperature was cooled to 0 °C, and H_2O (1.5 mL), aqueous 15% NaOH (1.5 mL), and H_2O (4.5 mL) were added to quench the reaction. After the celite filtration of the solution, the water in the solution was removed by $MgSO_4$, which was filtered and concentrated under reduced pressure to give 4-phenylbutan-1-ol (**1a-I**, 4.76 g, 31.7 mmol, 79% crude yield) as the crude material. This compound was identified by

the comparison of the reported spectroscopic data.[2] Because the purity of the crude material was high, synthesized 4-phenylbutan-1-ol (**1a-I**) was used in the next step without the further purification.

To the solution of PPh_3 (11.80 g, 45.0 mmol), I_2 (11.43 g, 45.0 mmol), and imidazole (4.44 g, 65.2 mmol) in CH_2Cl_2 (100 mL), was slowly added 4-phenylbutan-1-ol (**1a-I**, 4.47 g, ca. 29.8 mmol) at room temperature. The reaction mixture was stirred for 2 h. After quenching the reaction by the addition of saturated aqueous $Na_2S_2O_3$ (100 mL), the additional CH_2Cl_2 (50 mL) and brine (100 mL) were added and separated. The aqueous phase was extracted by CH_2Cl_2 (50 mL × 2), and the combined organic extract was washed by saturated aqueous NH_4Cl (100 mL), followed by dried over $MgSO_4$. After the filtration and the concentration under reduced pressure, the crude product thus obtained was purified by the flash chromatography (hexane/AcOEt = 3:1) to give (4-iodobutyl)benzene (**1a-II**, 7.55 g, 29.0 mmol, 97% yield). This compound was identified by the comparison of the reported spectroscopic data.[3]

To the solution of ethynyltrimethylsilane (4.86 g, 49.5 mmol) and DMPU (*N*,*N'*-dimethylpropyleneurea, 6.36 g, 49.6 mmol) in THF (50 mL) at -78 °C, was slowly added *n*-BuLi (1.57 M, 31 mL, ca. 48.7 mmol). The solution was stirred for 1 h at the same temperature. Then, a solution of (4-iodobutyl)benzene (**1a-II**, 7.12 g, 27.4 mmol) in THF (50 mL) was slowly added to the reaction mixture at −78 °C, and stirred for 0.5 h at −78 °C. Then, the mixture was additionally stirred at room temperature overnight. The reaction was quenched by the addition of saturated aqueous NH_4Cl (100 mL). The additional Et_2O (50 mL) was added and separated. The aqueous phase was extracted by Et_2O (50 mL x 2), and the combined organic extract was washed by brine (50 mL), followed by dried over Na_2SO_4. After the filtration and concentration under reduced pressure, the crude product was purified by the flash chromatography (hexane/AcOEt = 10:1) to give trimethyl(6-phenylhex-1-yn-1-yl)silane (**1a-III**, 5.49 g, 23.8 mmol, 87% yield). This compound was identified by the comparison of the reported spectroscopic data.[3]

Trimethyl(6-phenylhex-1-yn-1-yl)silane (**1a-III**, 5.47 g, 23.7 mmol) was added to the round flask. MeOH (100 mL) and aqueous 2 M NaOH (24 mL) was added, and then the solution was stirred at room temperature overnight. The reaction was quenched by the addition of aqueous 2M HCl (24 mL). The brine (50 mL) and Et_2O (100 mL) were added and separated. The aqueous phase was extracted by Et_2O (100 mL × 2), and the combined organic extract was washed by brine (100 mL), followed by dried over Na_2SO_4. After the filtration and concentration under reduced pressure, the crude product was purified by the flash chromatography (hexane/AcOEt = 60:1) to give hex-5-yn-1-ylbenzene (**1a**, 3.26 g, 20.6 mmol, 87% yield). This compound was identified by the comparison of the reported spectroscopic data.[3]

Synthesis of 10-butoxydec-1-yne (1g).

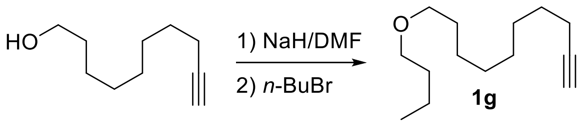

To the suspension of NaH (ca. 55% in mineral, 0.60 g, 13.8 mmol) in DMF (40 mL) at 0 °C, was slowly added dec-9-yn-1-ol (0.86 g, 5.6 mmol). Then, *n*-BuBr (1.28 g, 9.3 mmol) was added, and the reaction temperature was increased to room temperature. Then, the reaction mixture was stirred overnight. The reaction was quenched by the addition of H_2O (50 mL). CH_2Cl_2 (20 mL) and saturated aqueous NH_4Cl (20 mL) were added to the solution, and separated. The aqueous phase was extracted by CH_2Cl_2 (20 mL × 2), and the combined organic extract was washed by brine (80 mL × 2), followed by dried over $MgSO_4$. After the filtration and concentration under reduced pressure, the residue was filtered through a short column (9.4 × 3.2 cm) of silica gel by Et_2O to remove wastes to obtain the crude product. The crude product was purified by the first flash chromatography (hexane/AcOEt = 20:1) and second flash chromatography (hexane/AcOEt = 50:1) to give 10-butoxydec-1-yne (**1g**, 0.74 g, 3.5 mmol, 63% yield).

**10-butoxydec-1-yne (1g)** 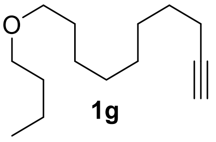 1g	**Unknown compound**; ^{1}H NMR (400 MHz, $CDCl_3$) δ 3.39 (t, *J* = 6.8 Hz, 2H), 3.34 (t, *J* = 6.4 Hz, 2H), 2.17 (td, *J* = 7.2, 2.8 Hz, 2H), 1.93 (t, *J* = 2.8 Hz, 1H), 1.62-1.47 (m, 7H), 1.44-1.18 (m, 9H), 0.91 (t, *J* = 7.6 Hz, 3H) ppm; ^{13}C NMR (100 MHz, $CDCl_3$) δ 84.8, 70.9, 70.6, 68.0, 31.8, 29.7, 29.3, 29.0, 28.7, 28.4, 26.1, 19.4, 18.4, 13.9 ppm; HRMS (ESI) calcd for $C_{14}H_{27}O$ ($[M+H]^+$): 211.2056, found: 211.2044.

Synthesis of 10-ididodec-1-yne (1i).

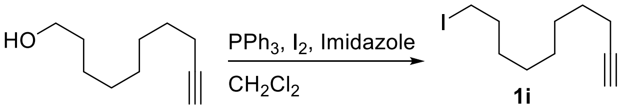

To the solution of PPh_3 (1.55 g, 5.9 mmol), I_2 (1.52 g, 6.0 mmol), and imidazole (0.67 g, 9.8 mmol) in CH_2Cl_2 (30 mL), was slowly added dec-9-yn-1-ol (0.66 g, 4.3 mmol) at room temperature. The solution was stirred at room temperature for 2 h. Then, the solution was refluxed for 1 h. The reaction was quenched by the addition of the saturated aqueous $Na_2S_2O_3$ (30 mL). CH_2Cl_2 (30 mL) was added to the solution and separated. The aqueous phase was extracted by CH_2Cl_2 (30 mL x 2), and the combined organic extract was washed by brine (30 mL), followed by dried over $MgSO_4$. After the filtration and the concentration under reduced pressure, the crude product was purified by the flash chromatography (hexane/AcOEt = 30:1) to give 10-iododec-1-yne (**1i**, 0.94 g, 3.6 mmol, 84% yield). This compound was identified by the comparison of the reported spectroscopic data.[4]

Typical procedure for the *gem*-difluorination in method A (Table 1, entry 2).

To the solution of Bu_4NBF_4 (823.0 mg, 2.5 mmol) in CH_2Cl_2 (4.0 mL), were added hex-5-yn-1-ylbenzene (**1a**, 78.6 mg, 0.497 mmol) and Tf_2NH (694.4 mg, 2.5 mmol). The reaction was stirred for 16 h at room temperature. Then, the reaction mixture was cooled to 0 °C and Et_3N (1.0 mL) was added to quench the reaction. After the concentration of the solution under reduced pressure, the residue was quickly filtered through a short column (9.4 × 3.2 cm) of silica gel by Et_2O to remove Bu_4NBF_4 and others. The silica gel was washed with Et_2O. The crude product was evaluated by ^{19}F NMR using trifluorotoluene ($CF_3C_6H_5$) (34.5 mg, 0.236 mmol) as an internal standard, indicating (5,5-difluorohexyl)benzene (**2a**) was obtained in 83% yield. Then, the crude product was purified via the flash chromatography (hexane) to give (5,5-difluorohexyl)benzene (**2a**, 71.1 mg, 0.359 mmol, 72% yield). In this step, ^{19}F NMR was also measured to confirm the absence of trifluorotoluene. This compound was identified by the comparison of the reported spectroscopic data.[5]

Other entries in Tables 1 and 2 were conducted according to the above procedure. If necessary, the preparative GPC separation was carried out for the crude products to purify and isolate the desired products.

Typical procedure for the *gem*-difluorination in method B (Scheme 1, 16 mA).

The electrochemical oxidation was carried out in an H-type divided cell (4G glass filter), which was equipped with a carbon felt anode and a platinum plate cathode (20 mm × 20 mm). In the cathodic and anodic chambers, 0.3 M Bu_4NBF_4/CH_2Cl_2 (8.0 mL × 2) was added. Then, hex-5-yn-1-ylbenzene (**1a**, 79.4 mg, 0.502 mmol) was added to the anodic solution. The constant current electrolysis (16 mA, 3.0 F/mol based on **1a**) at room temperature was conducted. After the consumption of the electricity, Et_3N (1.0 mL × 2) was added to both chambers to quench the reaction. After the concentration of the solution under reduced pressure, the residue was quickly filtered through a short column (9.4 × 3.2 cm) of silica gel by Et_2O to remove Bu_4NBF_4 and others. The crude product was evaluated by ^{19}F NMR using trifluorotoluene ($CF_3C_6H_5$) (33.4 mg, 0.229 mmol) as an internal standard, indicating (5,5-difluorohexyl)benzene (**2a**) was obtained in 42% yield. Then, the obtained crude product was purified

by the preparative GPC separation to give (5,5-difluorohexyl)benzene (**2a**, 29.0 mg, 0.146 mmol, 29% yield). In this step, ^{19}F NMR was also measured to confirm the absence of trifluorotoluene. This compound was identified by the comparison of the reported spectroscopic data.[5]

(3,3-Difluorobutyl)benzene (**2b**)[6] and 2-(4,4-difluoropentyl)isoindoline-1,3-dione (**2h**)[7] were fully identified by the comparison of the reported spectroscopic data.

The identification of other compounds such as 2,2-difluorodecane (**2d**), 2,2-difluorooctadecane (**2e**), 9,9-difluorodecan-1-ol (**2f**), 1-butoxy-9,9-difluorodecane (**2g**), 9,9-difluoro-1-iodododecane (**2i**), and 6,6-difluorododecane (**2j**) are described below. These compounds are either new compounds or known compounds but their spectra are not fully reported. Therefore, we report them in this paper. The analysis of ^{13}C NMR of these compounds was according to the literature, in which the analysis of ^{13}C NMR of 2,2-difluorotridecane is described.[8]

**2,2-difluorodecane (2d)** 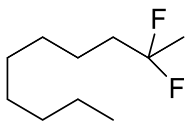 **2d**	**Known compound, but insufficient report for identification in some literatures (Table 2, ref 54)**; ^{1}H NMR (400 MHz, $CDCl_3$) δ 1.90-1.74 (m, 2H), 1.58 (t, *J* = 18.4 Hz, 3H), 1.50-1.40 (m, 2H), 1.38-1.18 (m, 10H), 0.88 (t, *J* = 6.8 Hz, 3H) ppm; ^{13}C NMR (100 MHz, $CDCl_3$) δ 124.5 (t, *J* = 236.5 Hz), 37.9 (t, *J* = 25.7 Hz), 31.8, 29.3, 29.3 (overlapping), 29.1, 23.2 (t, *J* = 28.6 Hz), 22.7 (t, *J* = 4.8 Hz), 22.6, 14.1 ppm; ^{19}F NMR (376 MHz, $CDCl_3$) δ from -90.17 to -90.43 (m, 2F) ppm; GC-MS (EI) m/z (%) = 158 (1) ($[M-HF]^+$), 138 (6), 65 (61), 43 (100). This compound was not detected by HRMS (ESI). The related aliphatic difluorinated compound such as 2,2-difluorododecane was detected by GC-MS in the literature,[9] in which $[M-HF]^+$ was confirmed. Because of the reason, we also carried out the analysis of GC-MS for **2d**, as described above.
**2,2-difluorooctadecane (2e)** 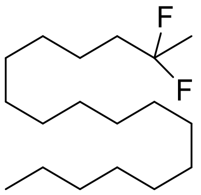 **2e**	**Unknown compound (Table 2, entry 8)**; ^{1}H NMR (400 MHz, $CDCl_3$) δ 1.89-1.71 (m, 2H), 1.57 (t, *J* = 18.2 Hz, 3H), 1.50-1.38 (m, 2H), 1.37-1.18 (m, 26H), 0.88 (t, *J* = 6.8 Hz, 3H) ppm; ^{13}C NMR (100 MHz, $CDCl_3$) δ 124.4 (t, *J* = 236.5 Hz), 38.0 (t, *J* = 24.8 Hz), 31.9, 29.7, 29.6, 29.5, 29.41, 29.38, 29.36, 23.2 (t, *J* = 27.6 Hz), 22.8 (t, *J* = 4.7 Hz), 22.7, 14.1 ppm. Some signals of ^{13}C NMR seemed to be overlapping. ^{19}F NMR (376 MHz, $CDCl_3$) δ from -90.15 to -90.42 (m, 2F) ppm; GC-MS (EI) m/z (%) = 270 (3) ($[M-HF]^+$), 250 (39), 71 (67), 57 (100), 43 (70). This compound was not detected by HRMS (ESI). The related aliphatic difluorinated compound such as 2,2-difluorododecane was detected by GC-MS in the literature,[9] in which $[M-HF]^+$ was confirmed. Because of the reason, we also carried out the analysis of GC-MS for **2e**, as described above.
**9,9-difluorodecan-1-ol (2f)** 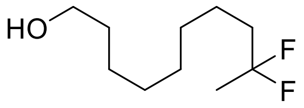 **2f**	**Unknown compound (Table 2, entry 10)**; ^{1}H NMR (400 MHz, $CDCl_3$) δ 3.64 (t, *J* = 6.4 Hz, 2H), 1.90-1.74 (m, 2H), 1.64-1.51 (m, 5H), 1.51-1.41 (m, 2H), 1.41-1.26 (m, 9H) ppm; ^{13}C NMR (100 MHz, $CDCl_3$) δ 124.4 (t, *J* = 235.5 Hz), 63.0, 37.9 (t, *J* = 24.8 Hz), 32.7, 29.3, 29.2, 29.2 (overlapping), 25.7, 23.2 (t, *J* = 28.6 Hz), 22.7 (t, *J* = 4.8 Hz) ppm; ^{19}F NMR (376 MHz, $CDCl_3$) δ from -90.18 to -90.45 (m, 2F) ppm; HRMS (ESI) calcd for $C_{10}H_{20}OF_2Na$ ($[M+Na]^+$): 217.1374, found: 217.1369.

**1-butoxy-9,9-difluorodecane (2g)**<br>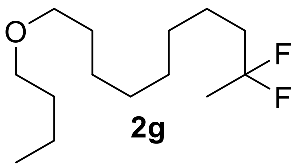<br>**2g**	**Unknown compound (Table 2, entry 12)**; ^{1}H NMR (400 MHz, $CDCl_3$) δ 3.40 (t, *J* = 6.4 Hz, 2H), 3.39 (t, *J* = 6.4 Hz, 2H), 1.90-1.74 (m, 2H), 1.63-1.51 (m, 7H), 1.50-1.25 (m, 12H), 0.92 (t, *J* = 7.2 Hz, 3H) ppm; ^{13}C NMR (100 MHz, $CDCl_3$) δ 124.4 (t, *J* = 236.5 Hz), 70.8, 70.6, 37.9 (t, *J* = 24.8 Hz), 31.8, 29.7, 29.29, 29.26, 29.22, 26.1, 23.1 (t, *J* = 27.7 Hz), 22.7 (t, *J* = 4.8 Hz), 19.3, 13.9 ppm; ^{19}F NMR (376 MHz, $CDCl_3$) δ from -90.18 to -90.46 (m, 2F) ppm; HRMS (ESI) calcd for $C_{14}H_{28}OF_2Na$ ($[M+Na]^+$): 273.2000, found: 273.1996.
**9,9-difluoro-1-iodoodecane (2i)**<br>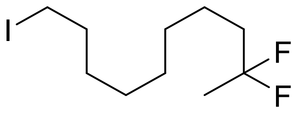<br>**2i**	**Unknown compound (Table 2, entry 16)**; ^{1}H NMR (400 MHz, $CDCl_3$) δ 3.19 (t, *J* = 6.8 Hz, 2H), 1.90-1.75 (m, 4H), 1.58 (t, *J* = 18.0 Hz, 3H), 1.51-1.27 (m, 10H) ppm; ^{13}C NMR (100 MHz, $CDCl_3$) δ 124.4 (t, *J* = 236.5 Hz), 37.9 (t, *J* = 24.8 Hz), 33.4, 30.4, 29.19, 29.16, 28.3, 23.2 (t, *J* = 28.6 Hz), 22.7 (t, *J* = 4.8 Hz), 7.3 ppm; ^{19}F NMR (376 MHz, $CDCl_3$) δ from -90.24 to -90.50 (m, 2F) ppm; HRMS (ESI) calcd for $C_{10}H_{19}F_2INa$ ($[M+Na]^+$): 327.0392, found: 327.0389.
**6,6-difluorododecane (2j)**<br>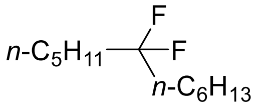<br>**2j**	**Known compound, but insufficient report for identification in the literature[10] (Table 2, entry 18)**;^{1}H NMR (400 MHz, $CDCl_3$) δ 1.87-1.71 (m, 4H), 1.50-1.40 (m, 4H), 1.38-1.21 (m, 10H), 0.94-0.85 (m, 6H) ppm; ^{13}C NMR (100 MHz, $CDCl_3$) δ 125.5 (t, *J* = 238.4 Hz), 36.29 (t, *J* = 25.7 Hz), 36.26 (t, *J* = 25.7 Hz), 31.6 (overlapping), 29.1, 22.5, 22.4, 22.3 (t, *J* = 4.7 Hz), 22.0 (t, *J* = 4.7 Hz), 14.0, 13.9 ppm; ^{19}F NMR (376 MHz, $CDCl_3$) δ -97.58 (quintet, *J* = 16.6 Hz, 2F) ppm; HRMS (ESI) calcd for $C_{12}H_{23}F_2$ ($[M-H]^-$): 205.1773, found: 205.1793.

References

1 Ashikari, Y.; Nokami, T.; Yoshida, J. *J. Am. Chem. Soc.* **2011**, *133*, 11840.

2 (a) Owen, C. P.; Shahid, I.; Olusanjo, M. S.; Patel, C. H.; Dhanani, S.; Ahmed, S. *J. Steroid Biochem. Mol. Biol.* **2008**, *111*, 117; (b) Yasuda, M.; Onishi, Y.; Ueba, M.; Miyai, T.; Baba, A. *J. Org. Chem.* **2001**, *66*, 7741.

3 Brutiu, B. R.; Bubeneck, W. A.; Cvetkovic, O.; Li, J.; Maulide, N. *Monats. Chem.* **2019**, *150,* 3.

4 Kojima, N.; Suga, Y.; Matsumoto, T.; Tanaka, T.; Akatsuka, A.; Yamori, T.; Dan, S.; Iwasaki, H.; Yamashita, M. *Bioorg. Med. Chem.* **2015**, *23,* 1276.

5 Wang, Z.; Guo, C. Y.; Yang, C.; Chen, J. P. *J. Am. Chem. Soc.* **2019**, *141*, 5617.

6 Dryzhakov, M.; Moran, J. *ACS Catal.* **2016**, *6*, 3670.

7 Lu, Z.; Bajwa, B. S.; Liu, S.; Lee, S.; Hammond, G. B.; Xu, B. *Green Chem.* **2019**, *21*, 1467.

8 Csuk, R.; Tamba, M. G.; Kluge, R. *Z. Naturforsch.* **2011**, *66b*, 1069.

9 Umemoto, T.; Singh, R. P.; Xu, Y.; Saito, N. *J. Am. Chem. Soc.* **2010**, *132*, 18199.

10 Farley, S. E. S.; Mulryan, D.; Rekhroukh, F.; Phanopoulos, A.; Crimmin, M. R. *Angew. Chem. Int. Ed.* **2024**, *63,* e202317550.

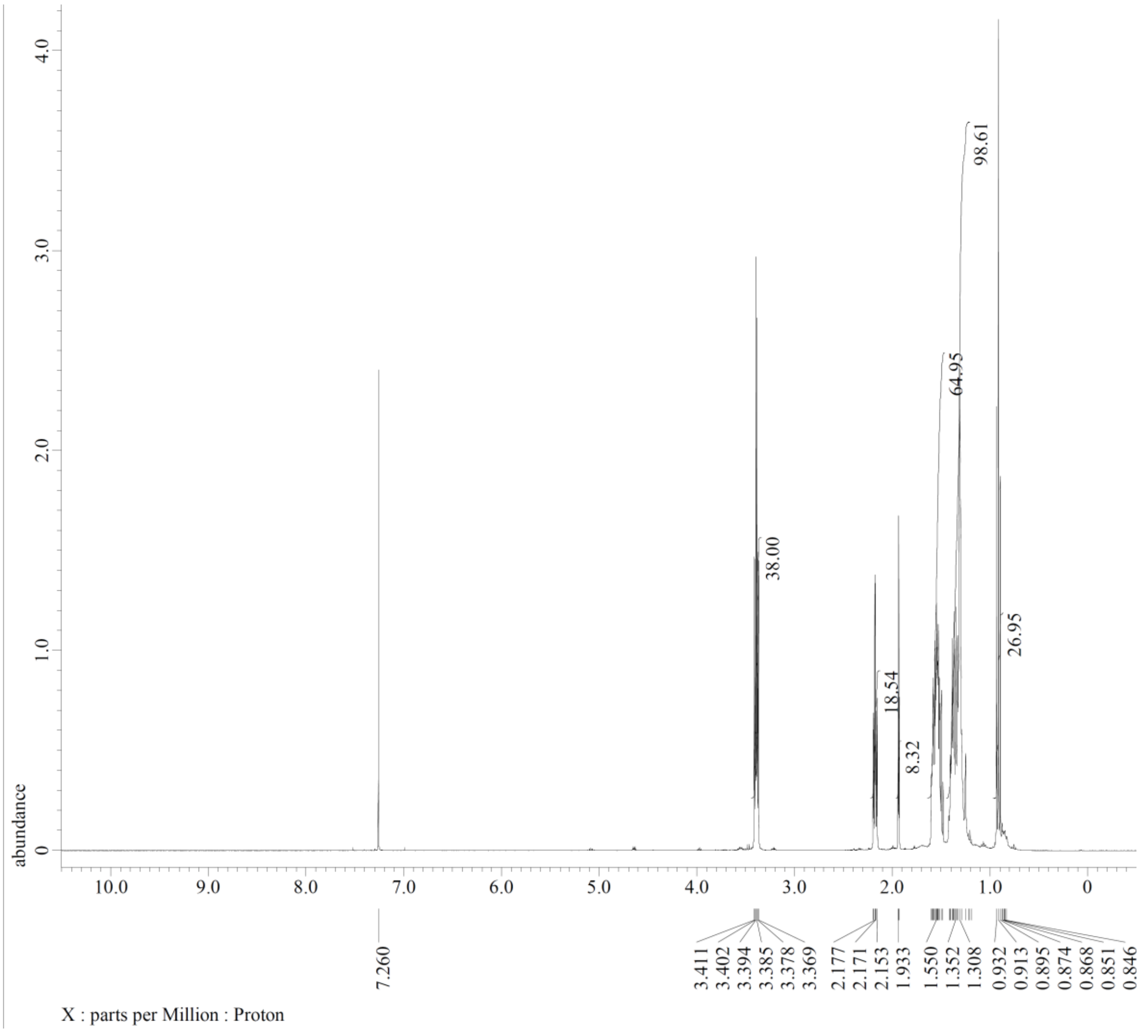

^{1}H NMR of 10-butoxydec-1-yne (**1g**).

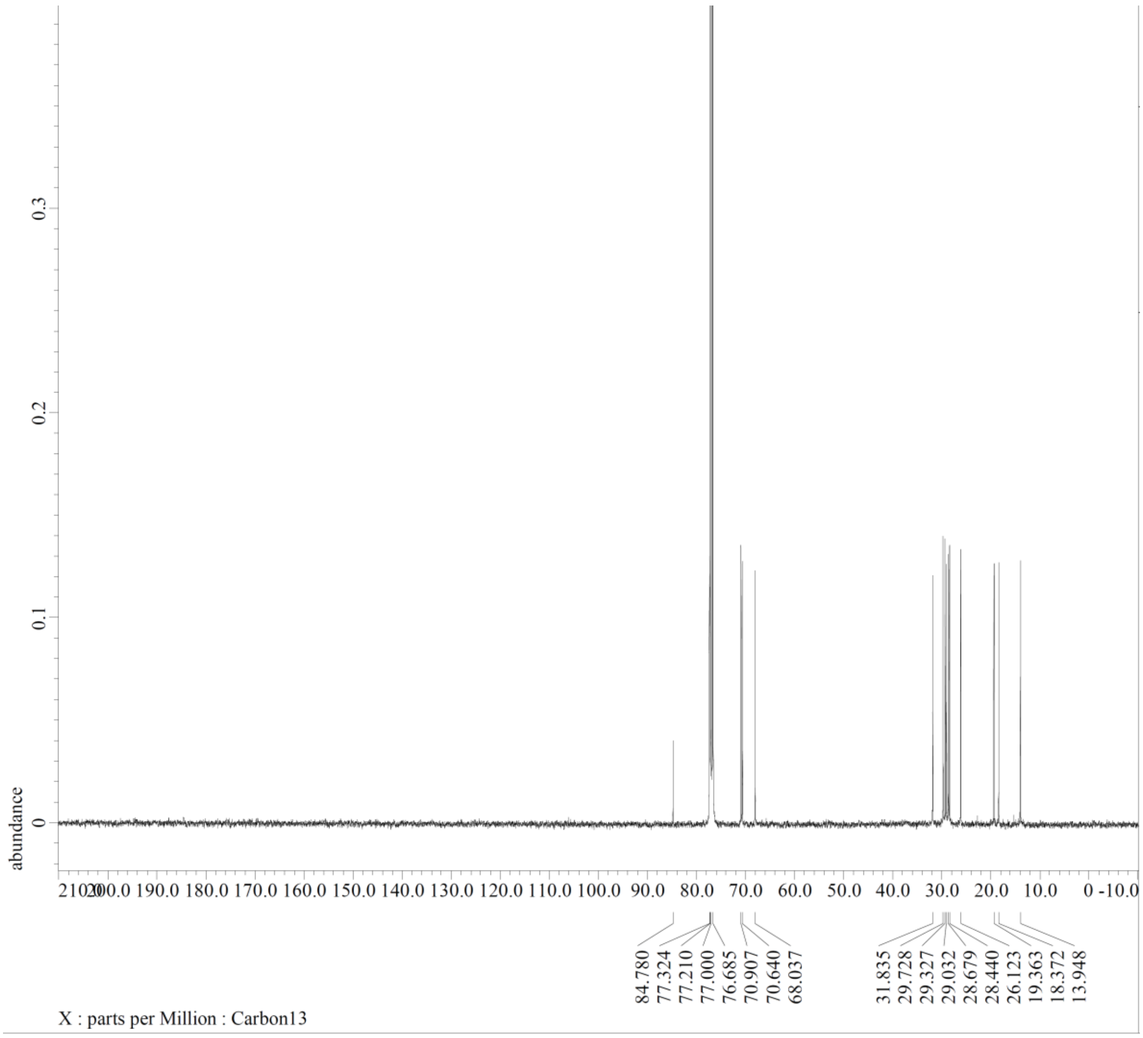

^{13}C NMR of 10-butoxydec-1-yne (**1g**).

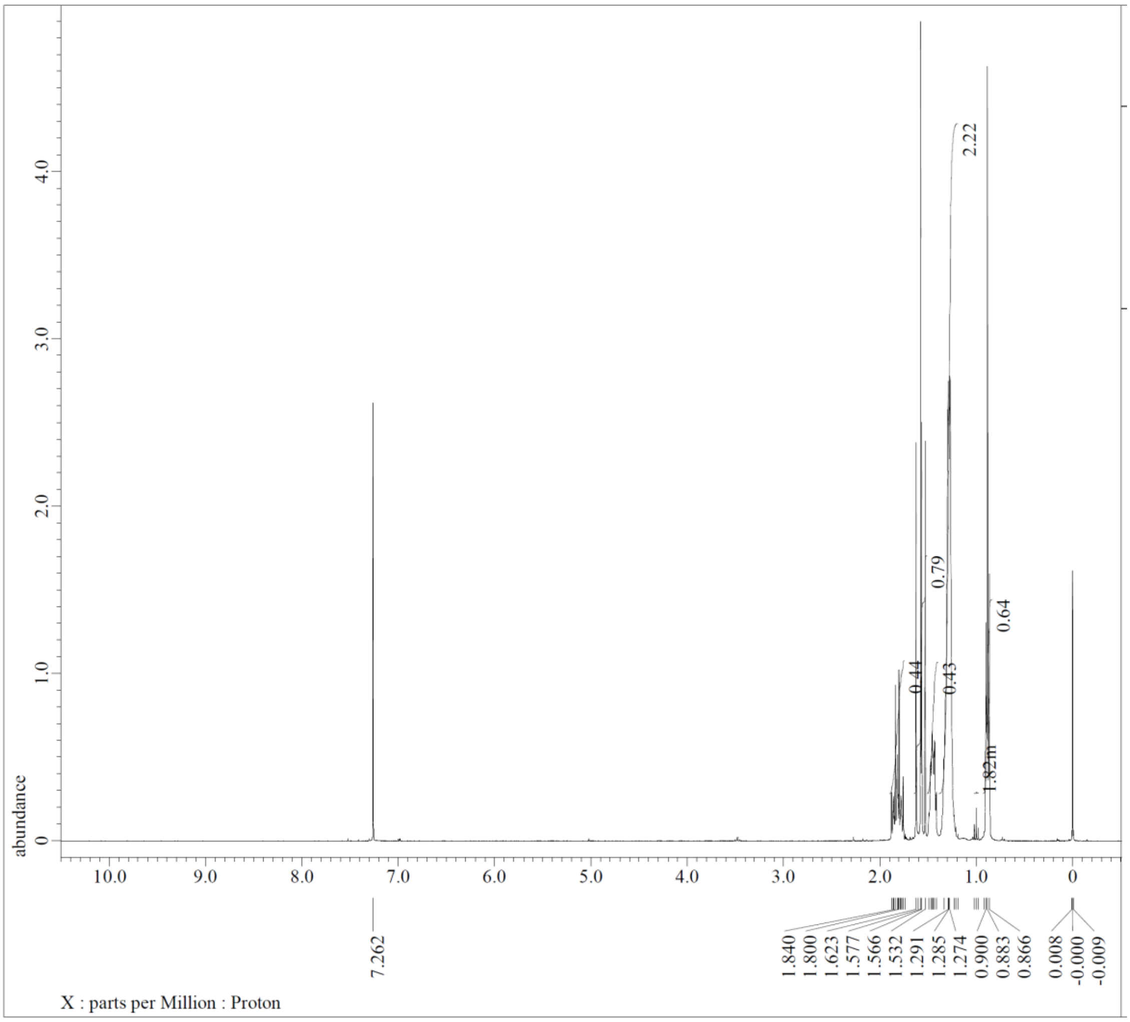

^{1}H NMR of 2,2-difluorodecane (**2d**).

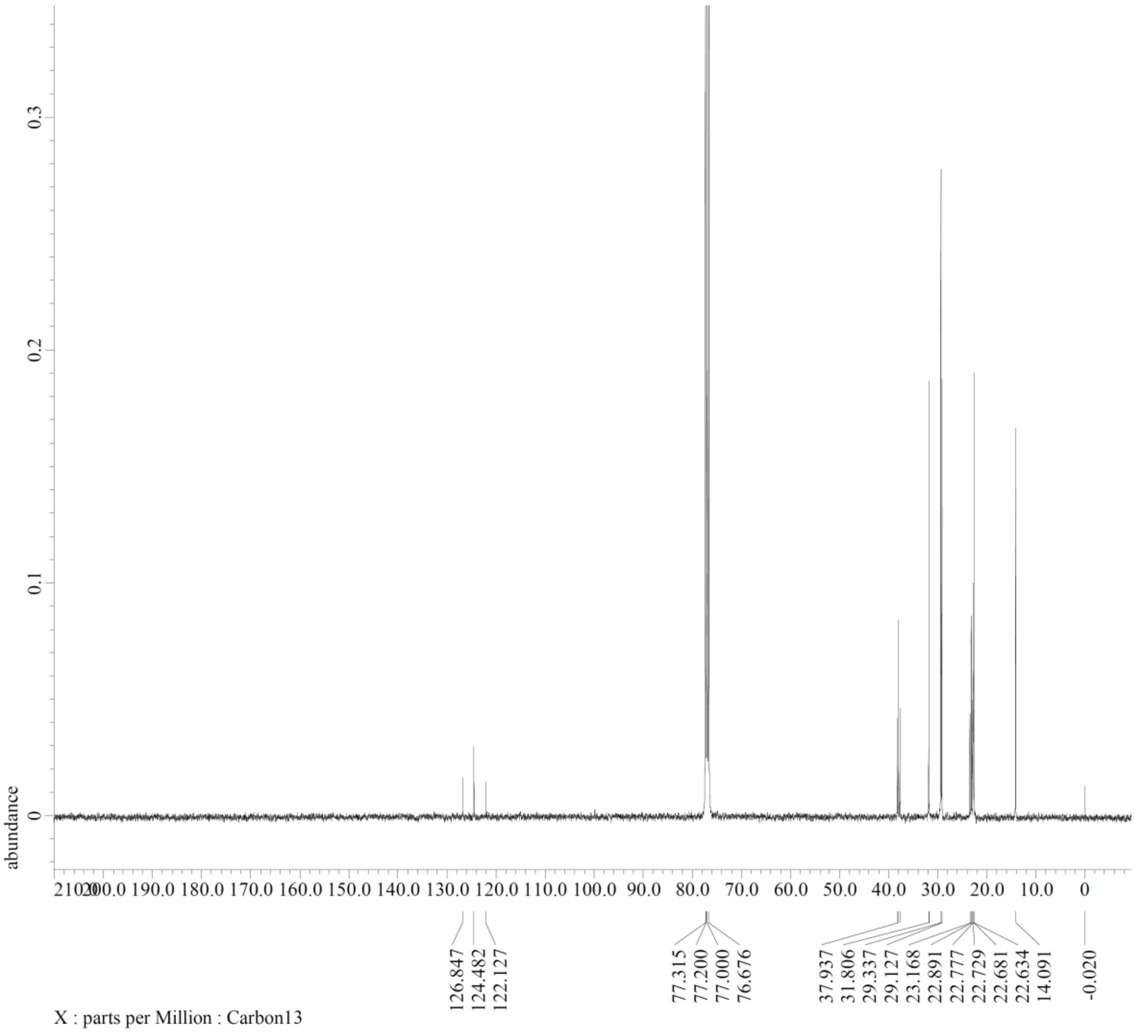

^{13}C NMR of 2,2-difluorodecane (**2d**).

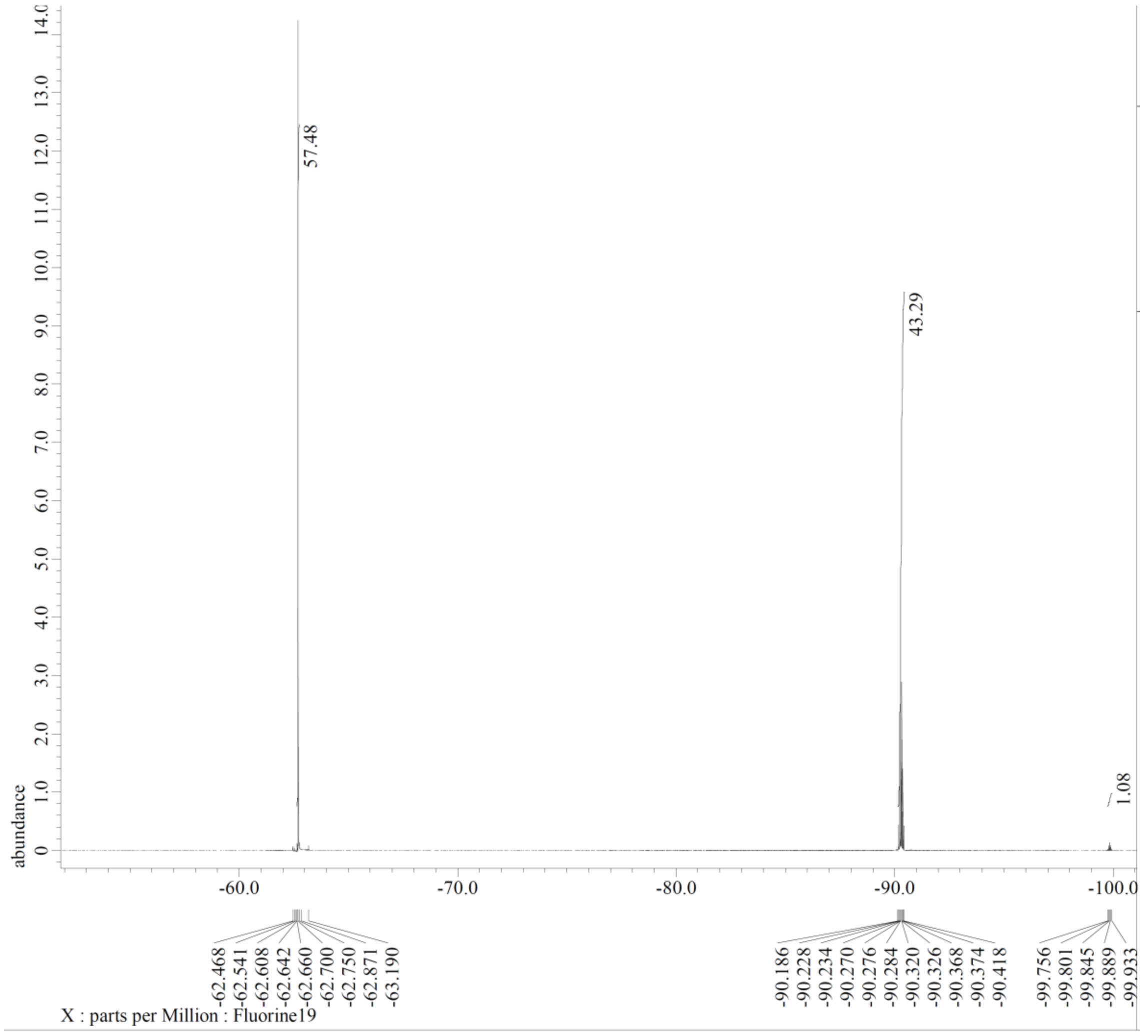

^{19}F NMR of 2,2-difluorodecane (**2d**) in the presence of $CF_3C_6H_5$ as an internal standard. This ^{19}F NMR spectrum was measured after the purification and isolation, adding $CF_3C_6H_5$.

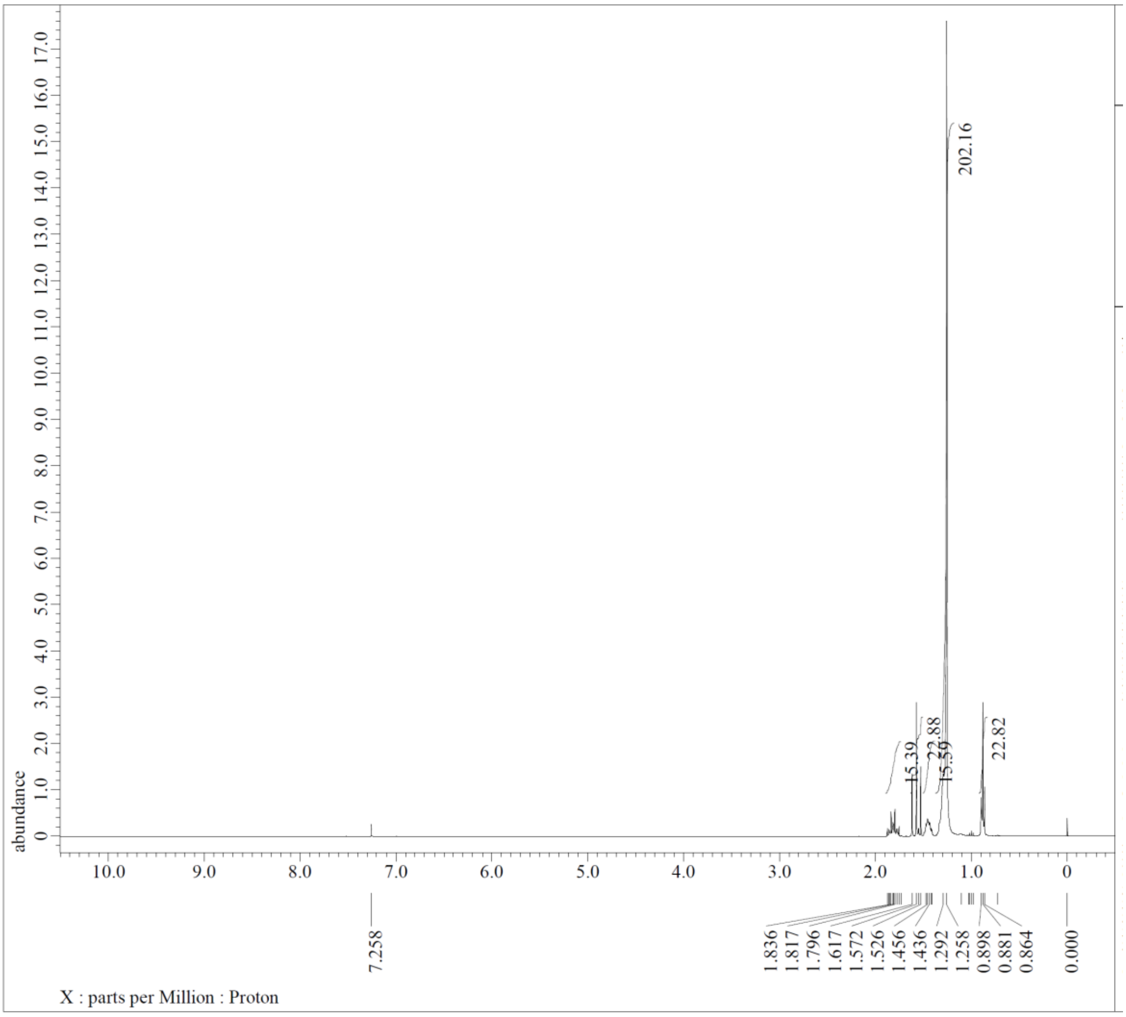

^{1}H NMR of 2,2-difluorooctadecane (**2e**).

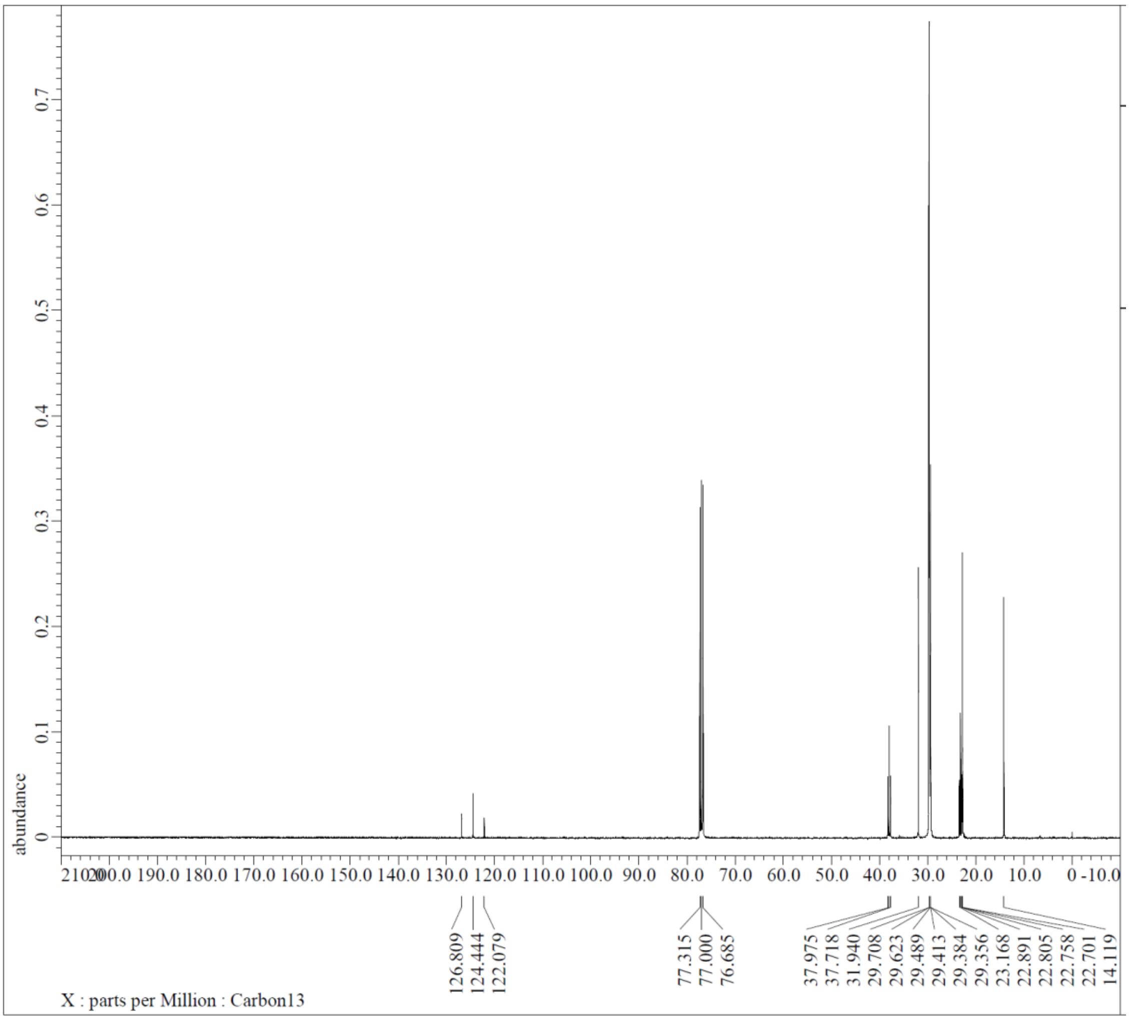

^{13}C NMR of 2,2-difluorooctadecane (**2e**).

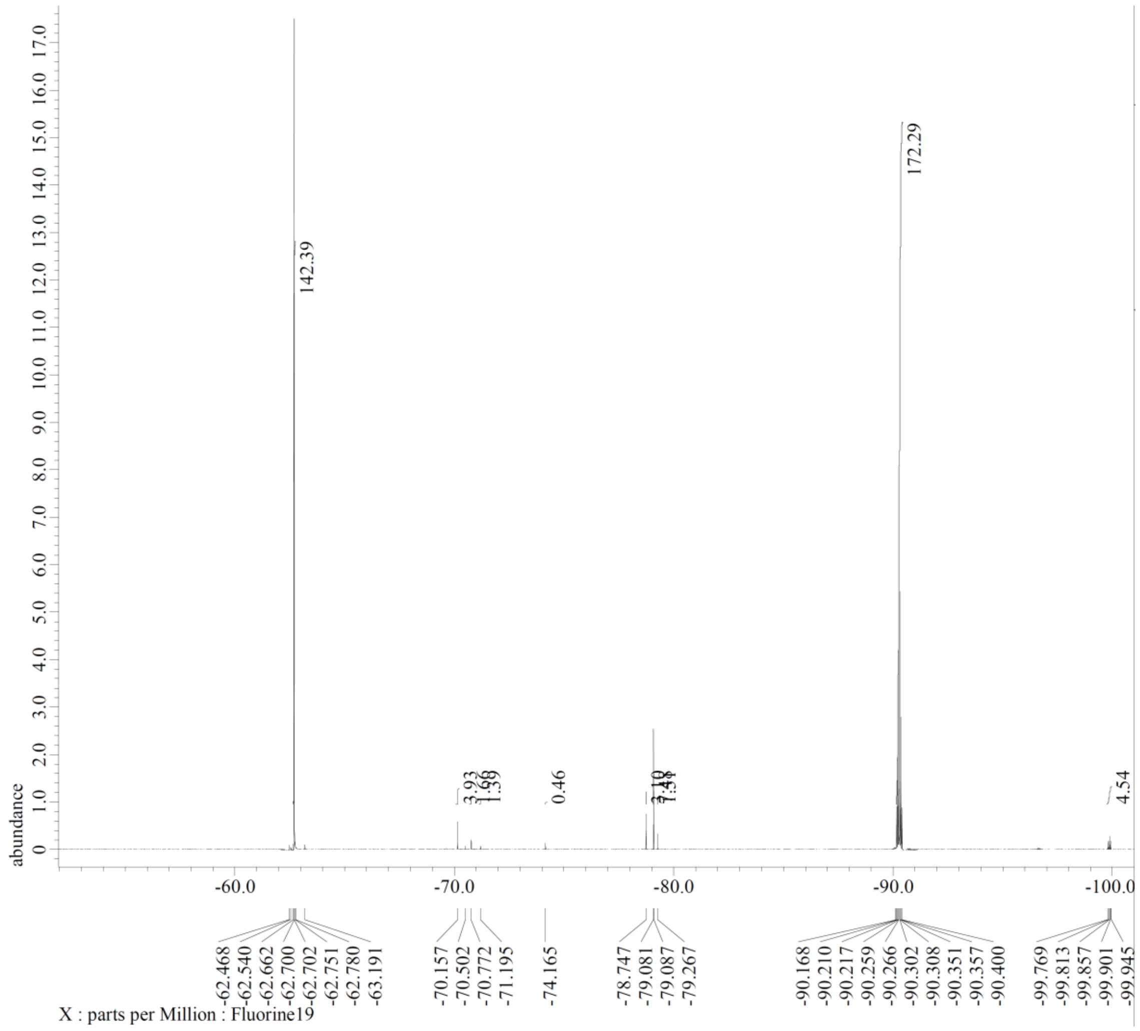

^{19}F NMR of 2,2-difluorooctadecane (**2e**) in the presence of $CF_3C_6H_5$ as an internal standard. This ^{19}F NMR spectrum was measured at the crude product.

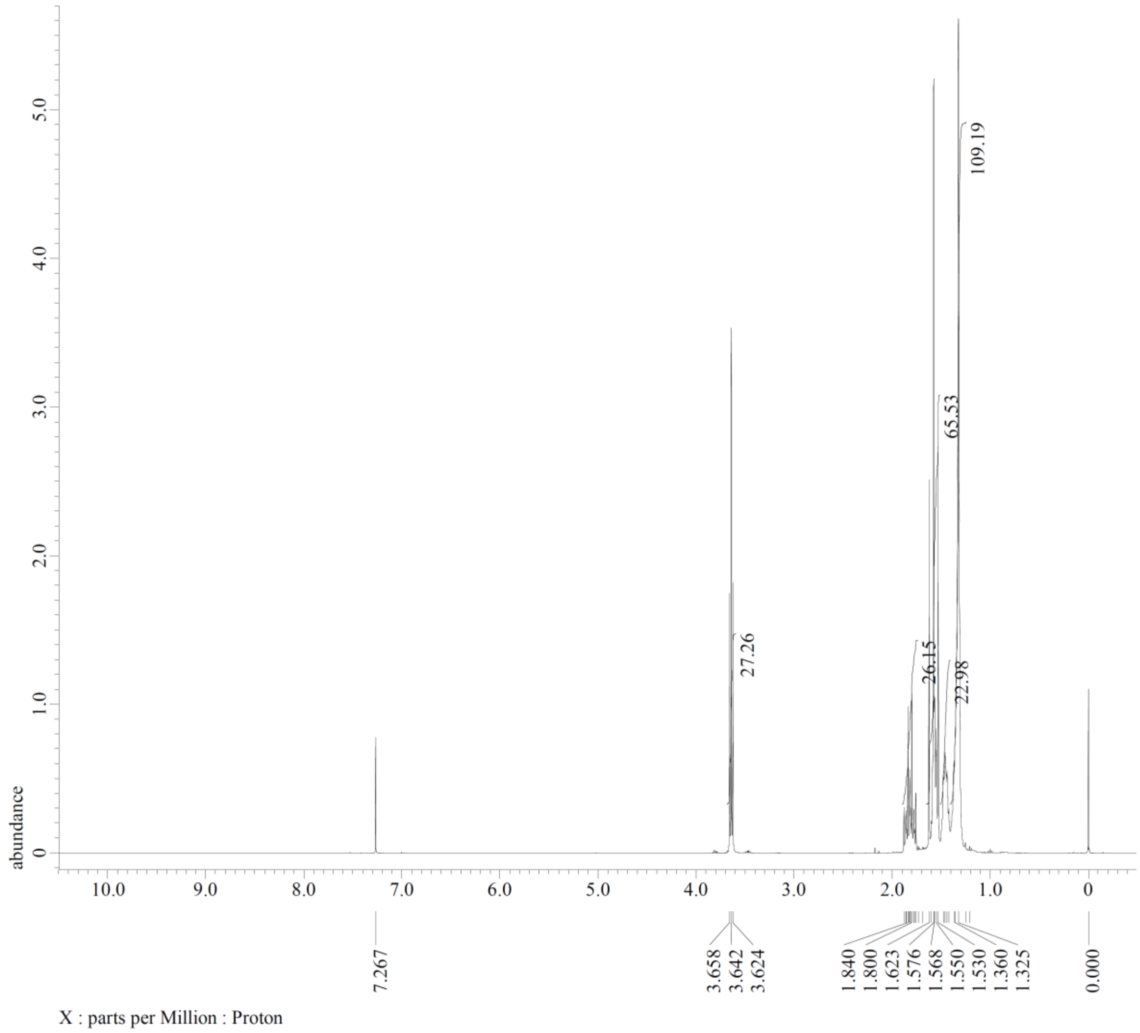

^{1}H NMR of 9,9-difluorodecan-1-ol (**2f**).

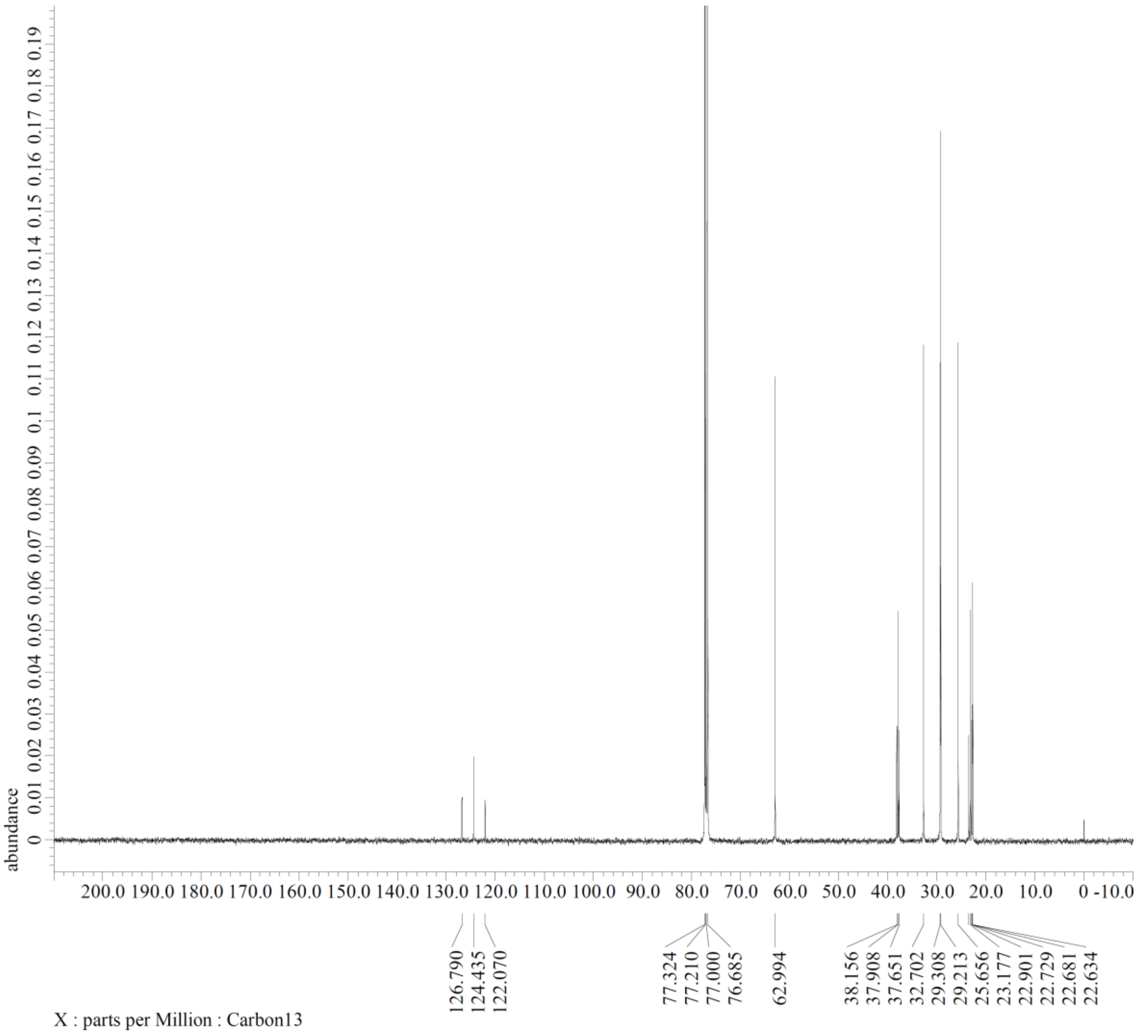

^{13}C NMR of 9,9-difluorodecan-1-ol (**2f**).

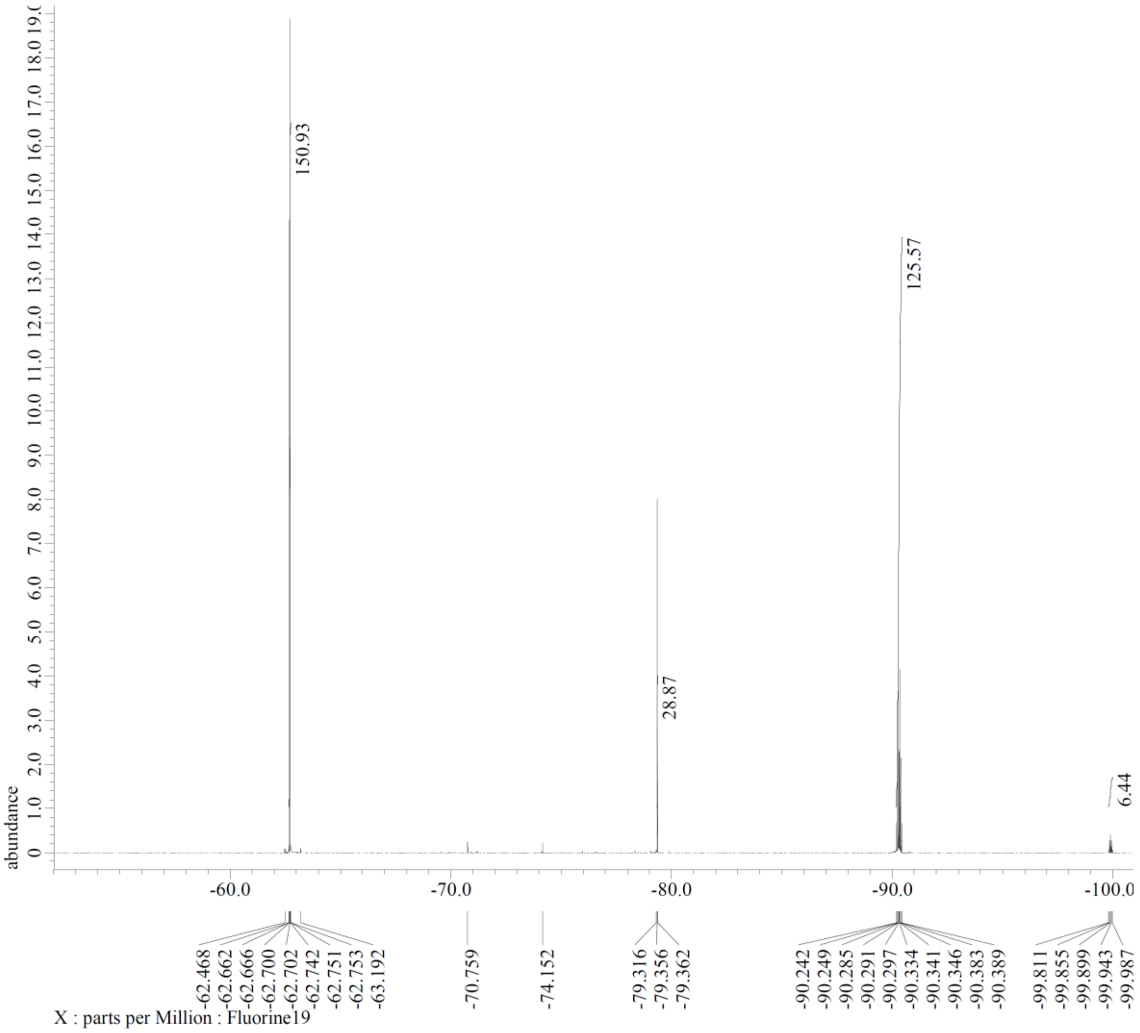

^{19}F NMR of 9,9-difluorodecan-1-ol (**2f**) in the presence of $CF_3C_6H_5$ as an internal standard. This ^{19}F NMR spectrum was measured at the crude product.

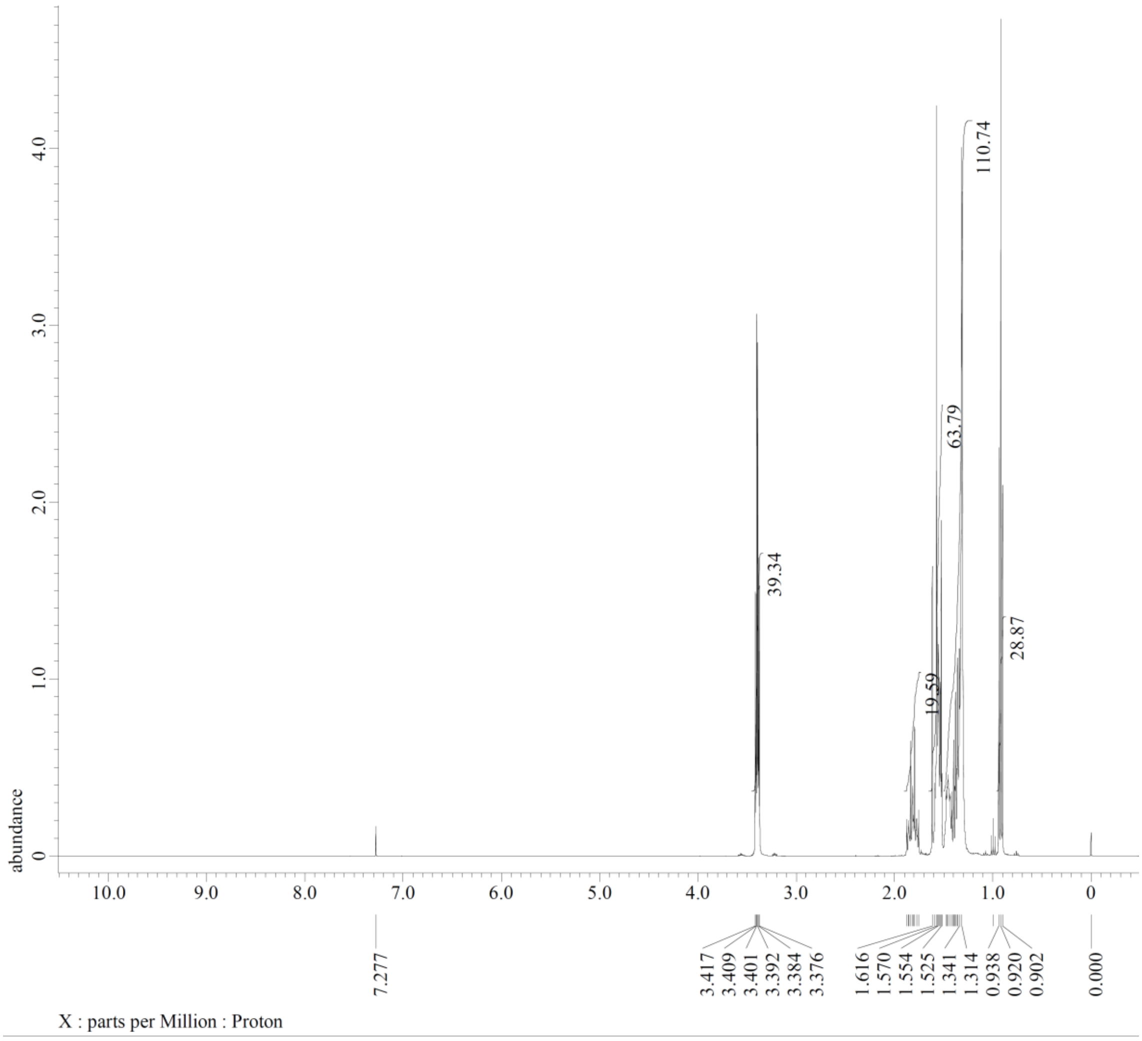

^{1}H NMR of 1-butoxy-9,9-difluorodecane (**2g**).

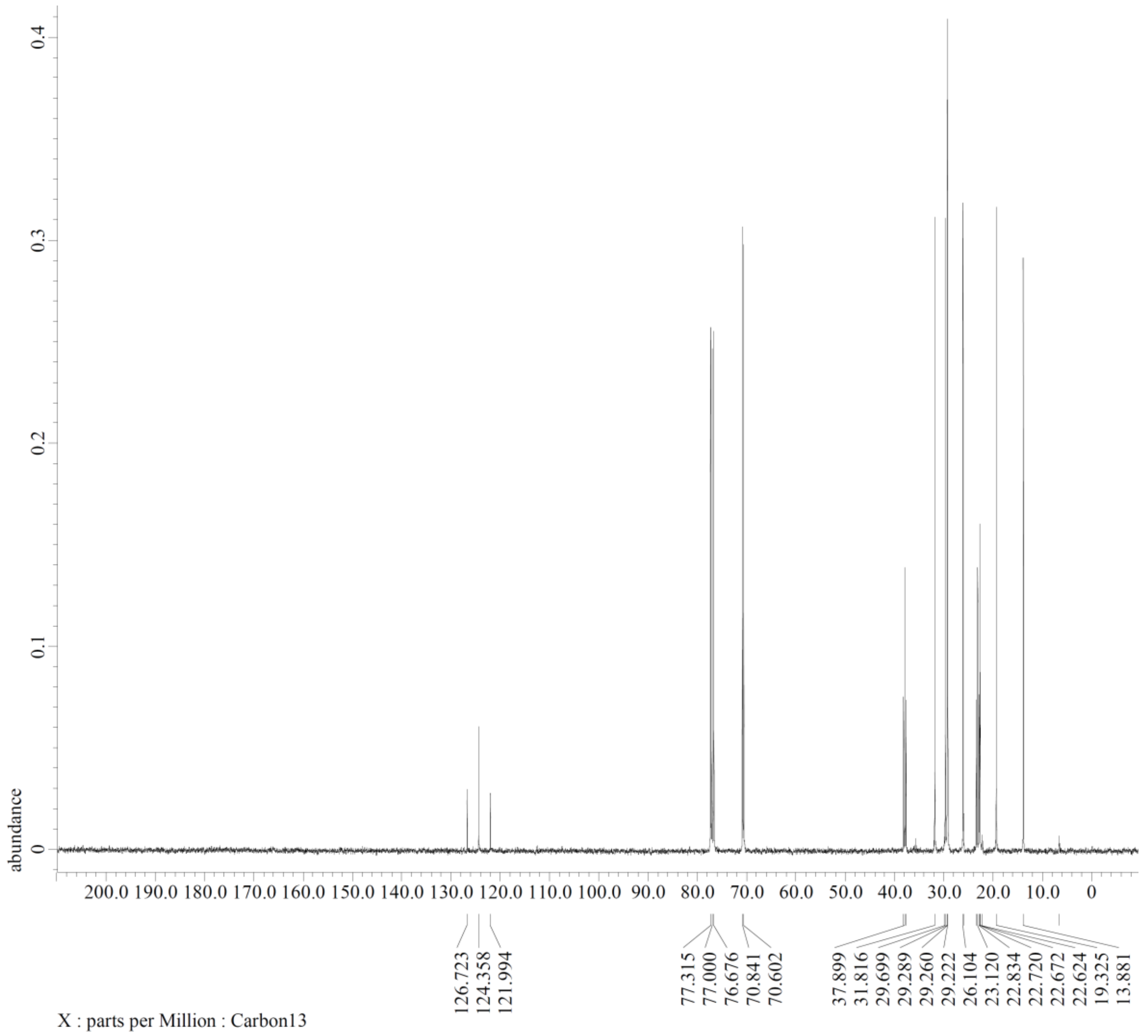

^{13}C NMR of 1-butoxy-9,9-difluorodecane (**2g**).

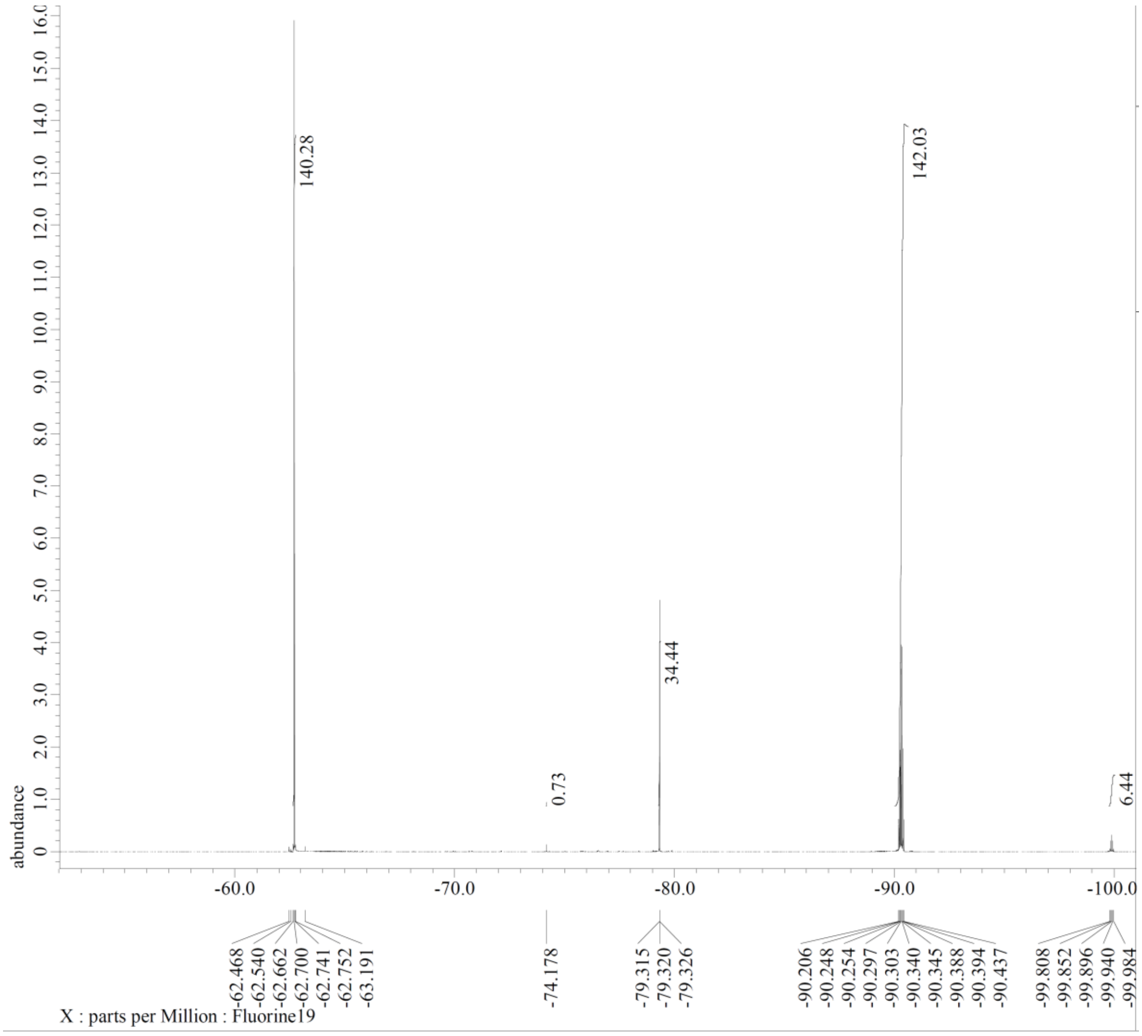

^{19}F NMR of 1-butoxy-9,9-difluorodecane (**2g**) in the presence of $CF_3C_6H_5$ as an internal standard. This ^{19}F NMR spectrum was measured at the crude product.

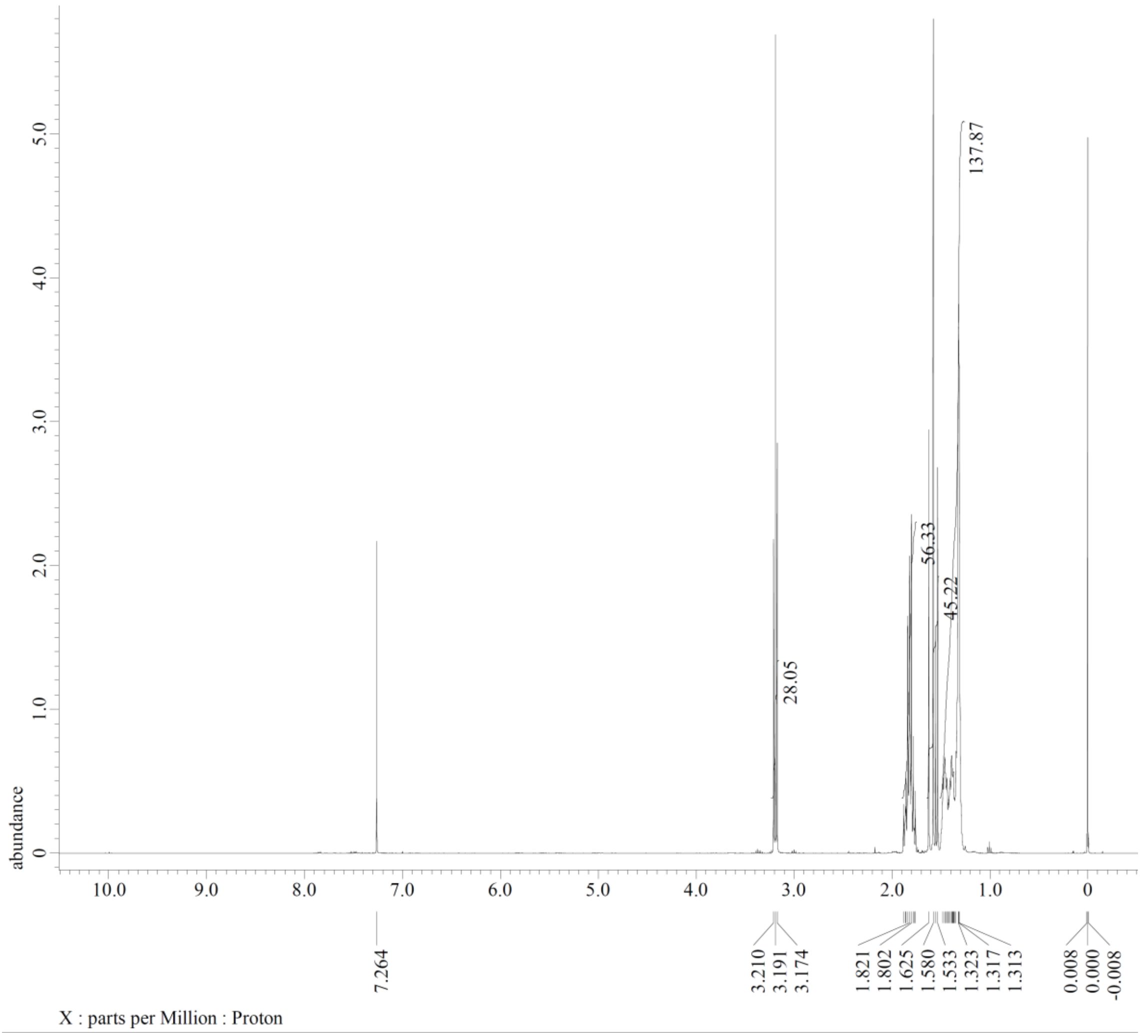

^{1}H NMR of 9,9-difluoro-1-iodododecane (**2i**).

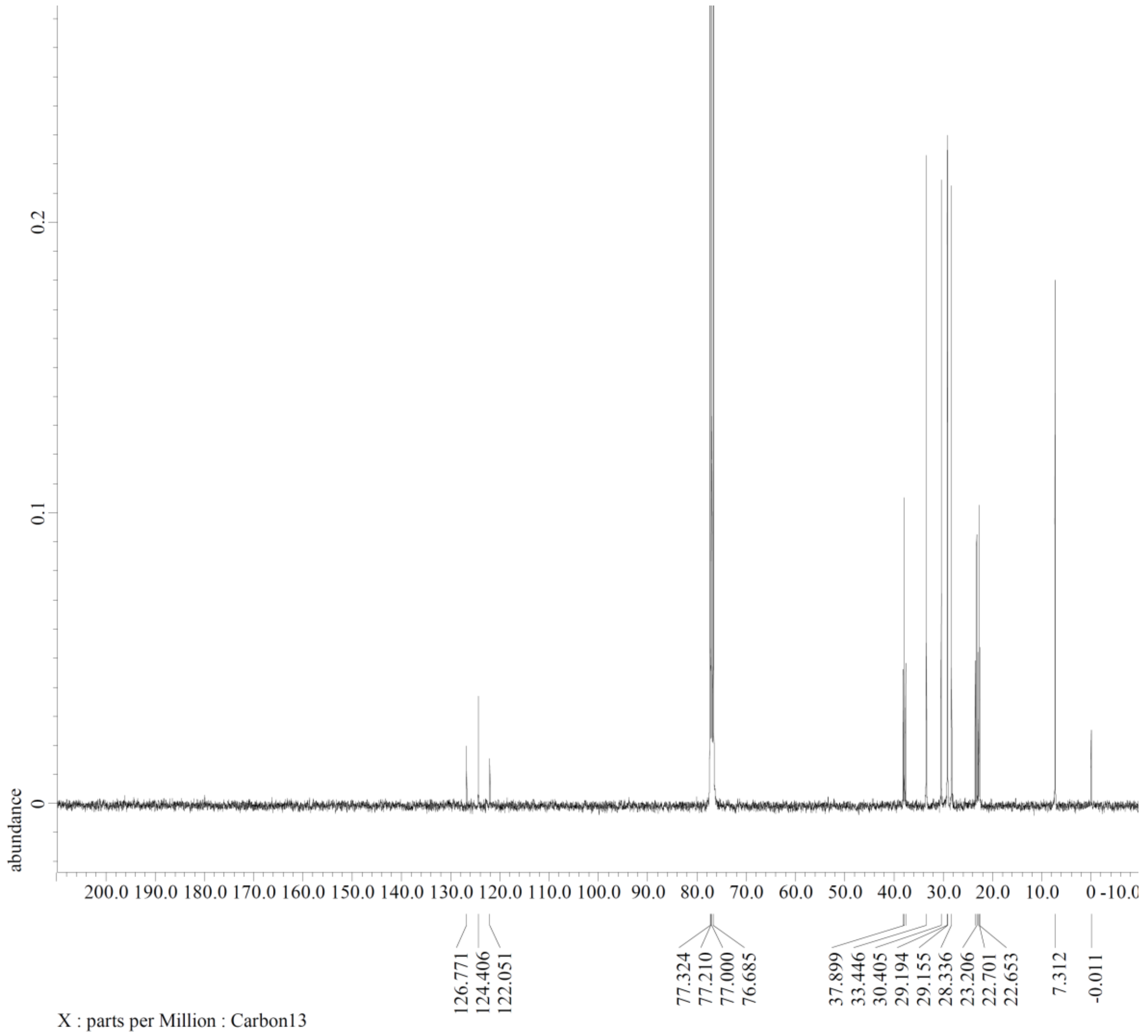

^{13}C NMR of 9,9-difluoro-1-iodododecane (**2i**).

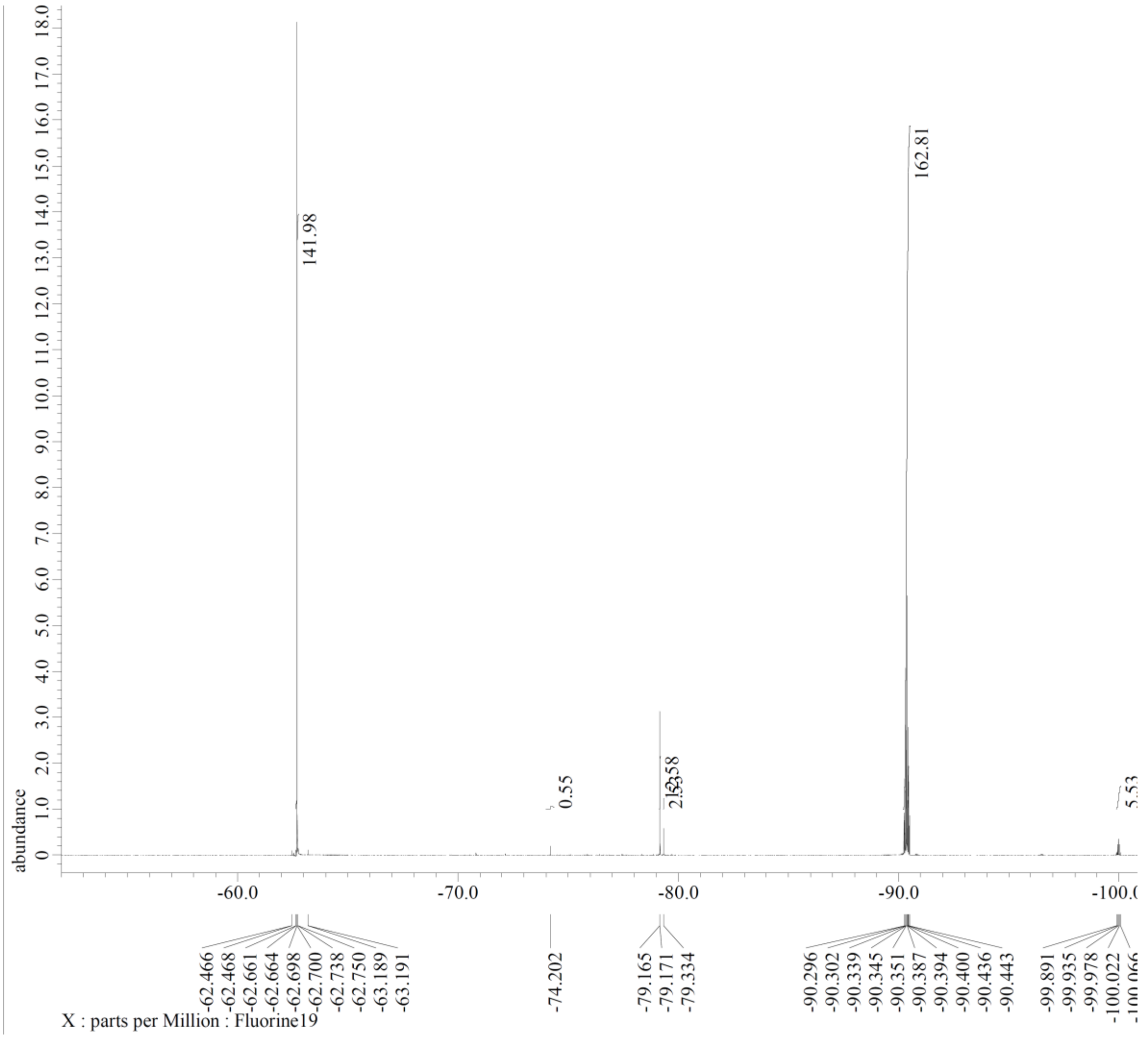

^{19}F NMR of 9,9-difluoro-1-iodododecane (**2i**) in the presence of $CF_3C_6H_5$ as an internal standard. This ^{19}F NMR spectrum was measured at the crude product.

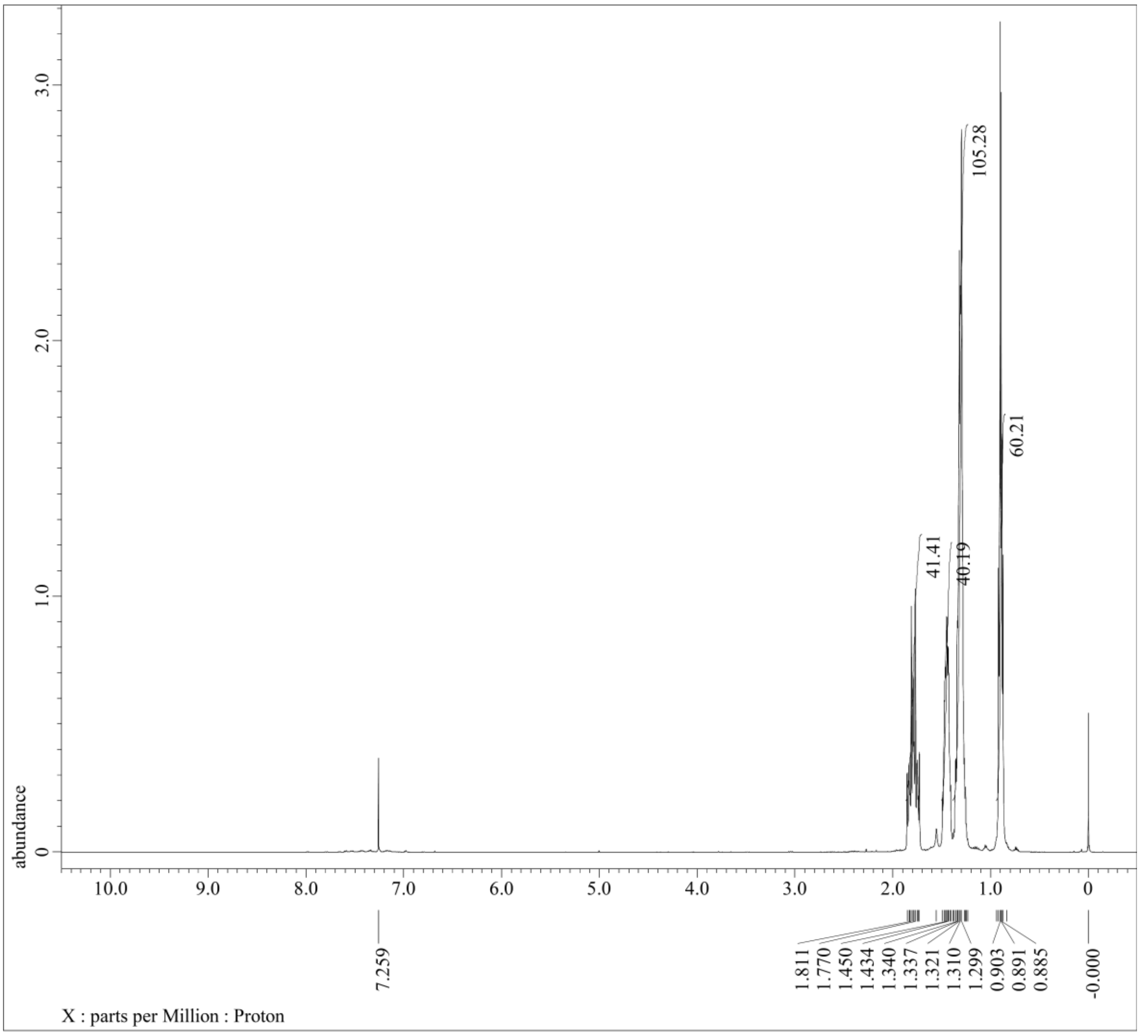

^{1}H NMR of 6,6-difluorododecane (**2j**).

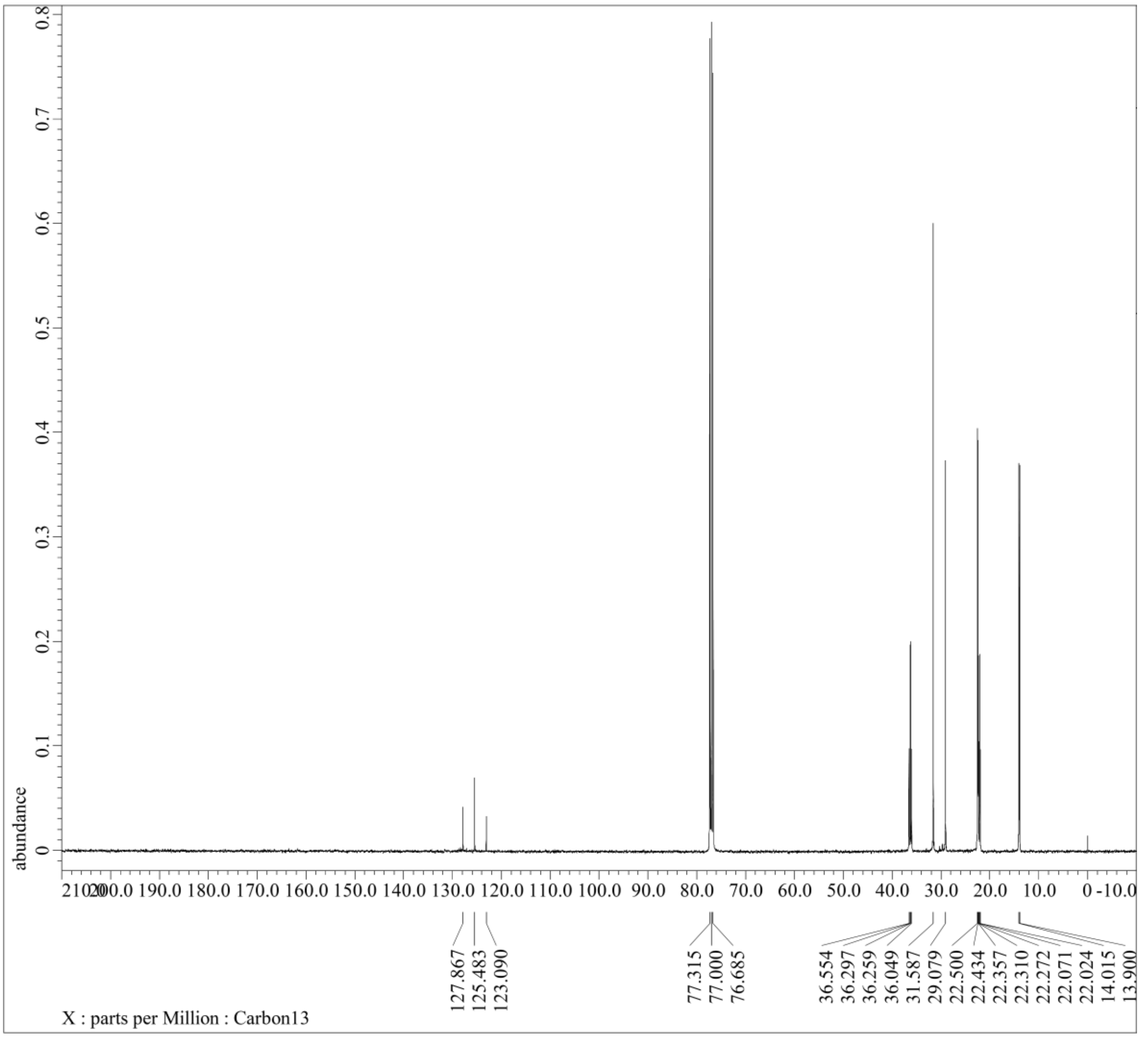

^{13}C NMR of 6,6-difluorododecane (**2j**).

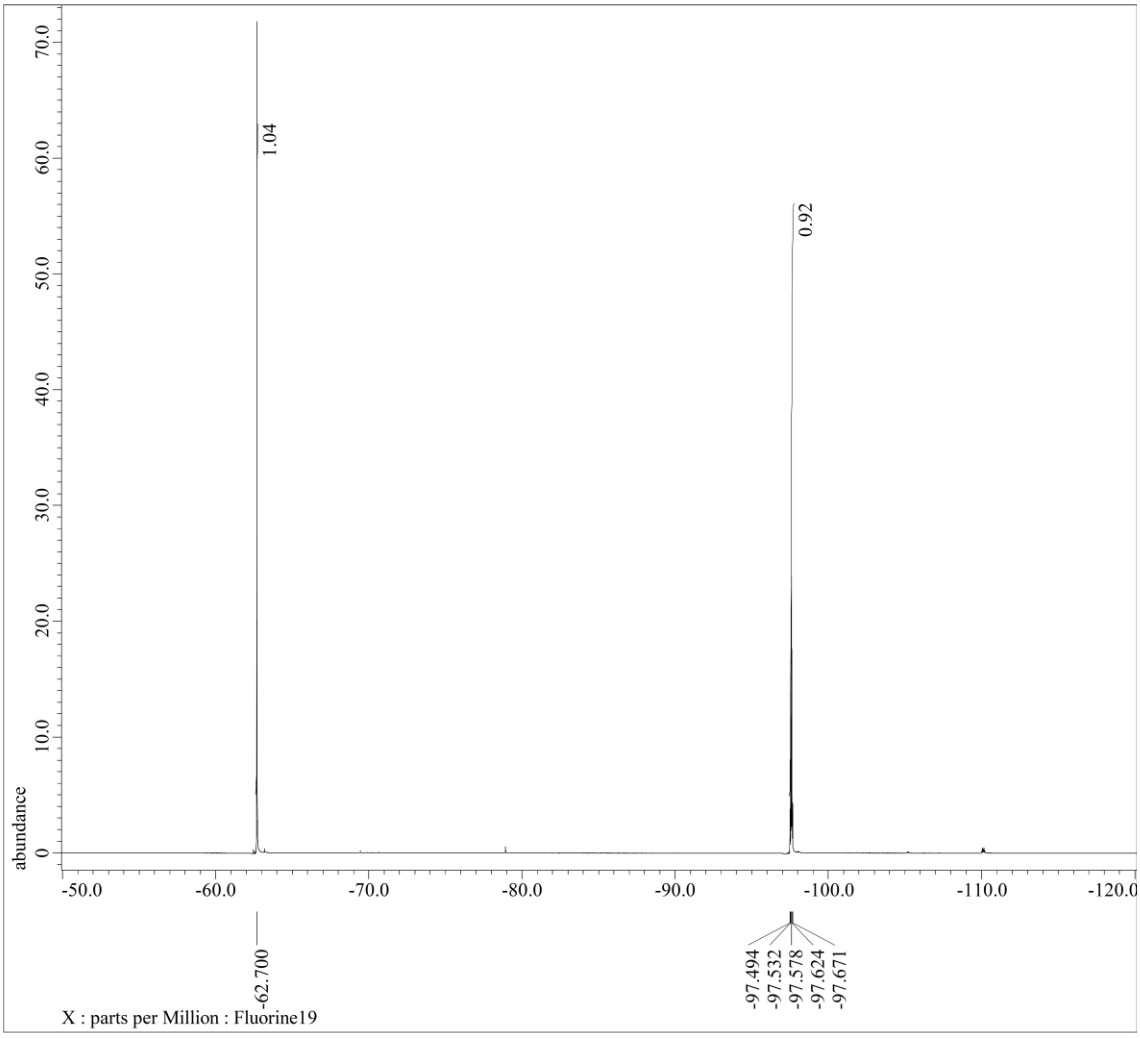

^{19}F NMR of 6,6-difluorododecane (**2j**) in the presence of $CF_3C_6H_5$ as an internal standard. This ^{19}F NMR spectrum was measured at the crude product.
